# Seasonal Analysis of Pathogenic *Escherichia coli* Contamination in Vegetables, Washing Water, and Vendor Hygiene: Virulence Group Classification and Antibiotic Resistance

**DOI:** 10.1002/fsn3.4723

**Published:** 2025-01-09

**Authors:** Maftuha Ahmad Zahra, Golam Niaj Murshidi, Unmilita Das Moon, Sumaiya Sultana, Fahim Kabir Monjurul Haque

**Affiliations:** ^1^ Microbiology Program, Department of Mathematics and Natural Sciences BRAC University Dhaka Bangladesh

**Keywords:** *E. coli* pathotypes, *Escherichia coli*, hand swabs, multidrug resistance, vegetables, wash water

## Abstract

This study, conducted between June 2022 and March 2023 in Dhaka, examined *Escherichia coli* prevalence in 874 samples from vegetables, vegetable wash water, and hand swabs from vendors during summer and winter. Of the total samples, 782 (89.50%) tested positive for *E. coli* , with 95.52% of samples in summer and 80.87% in winter. While overall *E. coli* prevalence showed no significant seasonal difference, pathotype prevalence was significantly higher in summer across all sample types, except for the *CVD432* gene. *E. coli* isolated from spring onions had the highest prevalence of *E. coli* O157:H7 (19.23%) and the *stx1* gene (30.76%), while capsicum isolates showed the highest prevalence of *stx2* (40.00%), *eaeA* (20.00%), *ipaH* (35.00%), and *eltB* (20.00%) genes. In winter, coriander had the highest *E. coli* O157:H7 (14.28%), and cucumber isolates had the highest *stx1* (19.04%) gene. Isolates from tomato and capsicum recorded elevated *stx2* levels (16.00%). Carrot isolates exhibited the highest *eaeA* prevalence (11.42%), coriander isolates had the highest *ipaH* occurrence (14.28%), and tomato isolates had the highest *eltB* levels (16.00%). A significant seasonal difference was observed in only the *stx1* gene, which was higher in summer for all vegetables. Antibiotic susceptibility testing of 1206 isolates revealed widespread resistance, particularly to ampicillin and erythromycin. Significant seasonal differences in resistance were noted in vegetable samples, but not in water and hand swab samples. Multidrug resistance was highest in isolates from spring onions (56.60% in summer) and carrots (71.87% in winter), with extensively drug‐resistant isolates highest in mint (2.17% in summer) and carrots (6.25% in winter).

## Graphical abstract

From 874 samples of vegetables, water, and hand swabs, 782 (89.47%) tested positive for *E. coli* , all exceeding permissible limits. The *E. coli* O157:H7 strain was found in 8.69% of the samples. STEC genes were detected, with stx1 in 16.87% and stx2 in 16.75% of samples. EPEC was present in 9.2%, EIEC in 14.32%, and ETEC in 10.1% of the samples. No EAEC was found. Multidrug resistance was observed in 45.6% of samples and extensive drug resistance in 0.55%.

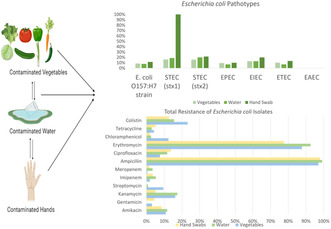

## 1 Introduction

Due to its numerous nutritional benefits, the consumption of fresh produce has witnessed a considerable increase in recent years. Research shows that global fresh vegetable consumption has surged 25.00% in last 30 years (Luna‐Guevara et al. 2019). Those with vegetable‐rich diets show resilience against cancer and heart disease (Boeing et al. 2012). Even though consuming vegetables has benefits, the consumption of bacterial‐contaminated fresh vegetables can lead to foodborne outbreaks. *Escherichia coli* , a prevalent bacterium, is commonly detected in such instances (Callejón et al. 2015). According to the Food Safety (Determination and Control of Microbiological Contaminants) Regulations of 2023 established by the Bangladesh Food Safety Authority (BFSA) (Sarker 2023), the presence of *E. coli* O157:H7 or Vero or Shiga toxin‐producing *E. coli* (STEC) in 25 g of cut or minimally processed and packed (non‐thermally processed) vegetables should be entirely absent. For ready‐to‐eat packaged fresh vegetables, the permissible level of *E. coli* is 10^1^–10^2^ CFU/g. According to the International Commission on Microbiological Specifications for Foods (ICMSF), acceptable *E. coli* levels in frozen fruits and vegetables (pH > 4.5) and dried vegetables are 10^2^–10^3^ CFU/g (Stewart 1987). The European Commission (EC) guidance document specifies that clean water, with an allowable *E. coli* level of 100 CFU/100 mL, is suitable for post‐harvest cooling and transportation of fresh fruits and vegetables that are not yet ready to eat. This limit is also deemed acceptable for the initial washing of products in the case of fresh produce that is ready to eat (Council of the European Union 2017).

*E. coli* encompasses various strains containing transportable biological components such as plasmids, transposons, bacteriophages, and pathogenicity islands. These components render certain strains highly pathogenic and toxic and cause gastrointestinal illnesses (Sethabutr et al. 1993). *E. coli* infections are conventionally categorized into six distinct pathotypes based on their pathogenicity profiles, encompassing virulence factors, clinical manifestations, and phylogenetic characteristics These types are enteropathogenic *E. coli* (EPEC), enterohemorrhagic *E. coli* (EHEC), enterotoxigenic *E. coli* (ETEC), enteroaggregative *E. coli* (EAEC), enteroinvasive *E. coli* (EIEC), and diffusely adherent *E. coli* (DAEC) (Kaper, Nataro, and Mobley 2004). In South Korea, an EPEC outbreak linked to cucumber tofu affected 13 people (Lim et al. 2020), while in Denmark, 88 individuals were sickened by EIEC from imported spring onions (Torpdahl et al. 2023). An EHEC outbreak in a South Korean preschool affected 104 people, mostly children, likely due to improperly stored food at temperatures above 10°C (Heo et al. 2023). ETEC has been found in foods such as kimchi in Korea (Cho et al. 2014) and basil in Norway (MacDonald et al. 2015). An outbreak of STEC in Germany, carrying EAEC genes, was traced to bean sprouts. The outbreak resulted in 3842 cases and 54 deaths (Hebbelstrup Jensen et al. 2014). The most recent *E. coli* outbreak associated with vegetables was in 2021, where the Centers for Disease Control and Prevention (CDC) reported *E. coli* O157:H7 outbreaks associated with baby spinach and packaged salads, which resulted in one fatality ( *E. coli Outbreak Linked to Baby Spinach* 2021; *E. coli Outbreak Linked to Packaged Salads* 2021).

The Advisory Committee on the Microbiological Safety of Foods (ACMSF) reported 531 cases of illness, including one fatality, linked to various foodborne diseases in the UK between 2008 and 2010 (O'Brien 2011). In Bangladesh, an estimated 30 million individuals suffer from *E. coli* ‐related foodborne diseases, as reported by Afzalur Rahman et al. 2018. A previous study conducted in the country study found that the prevalence of *E. coli* in raw salad vegetables was 13/266 (4.39%) (Nigad Nipa et al. 2011). In a separate investigation focused on salad vegetables in Dhaka city, *E. coli* was observed to have a high bacterial load ranging from 10^4^ to 10^8^ CFU/g, exceeding the permissible levels set by the BFSA (Rahman and Noor 2012). A similar study conducted in various regions of Bangladesh on different vegetables also revealed same *E. coli* levels (10^4^–10^8^ CFU/g) (Ohiduzzaman Islam et al. 2022). In the majority of cases, *E. coli* is most frequently detected in raw vegetables during the postharvest period, particularly in spring onions, cabbage, mint, and coriander. This occurrence can result from direct contamination of fresh vegetables or the proliferation of pathogens during the mid‐harvest period. Moreover, the water used for plant irrigation is identified as a potential source of contamination (Estarda‐Garcia et al. 2002).

*E. coli* can be the primary reason for many foodborne illnesses. According to the World Health Organization (WHO), *E. coli* is one of the most common foodborne pathogens, affecting millions of people annually, sometimes leading to severe or fatal outcomes (*Food Safety* 2024). Similarly, the CDC identify STEC as one of the most significant threats among foodborne illness, requiring hospitalization (“About Food Safety” 2024). STEC is responsible for diseases such as diarrhea, typhoid, urinary tract infections, respiratory illness, and bloodstream infections ( *E. coli Facts Sheet* 2018). *E. coli* O157: H7 can adhere to or become trapped within various parts of the plant, such as leaves, sprouts, and fruit, even after rigorous washing or disinfection procedures (Beuchat 1999; Franz et al. 2007; Jeter and Matthysse 2005; Solomon, Yaron, and Matthews 2002; Taormina and Beuchat 1999). ETEC is a significant contributor to infantile and traveler's diarrhea. ETEC can persist in diverse environments, including fresh vegetables, drinking water, irrigation water, and rivers. It has been observed that plant tissues with mechanical injuries, providing nutrients, serve as favorable environments for the rapid proliferation of enteric infections such as ETEC (Shaw et al. 2005). Consequently, these bacteria infect various vegetable plants, leading to contamination that ultimately enters the human body upon consumption, resulting in illnesses as mentioned above. In addition, detecting *E. coli* in food or water typically signifies recent fecal contamination or dissatisfying sanitary conditions within food processing facilities (Odonkor and Mahami 2020; Price and Wildeboer 2017). Fresh produce has a chance to become contaminated due to both the water used during the washing process and the handling practices applied by vendors (Desiree et al. 2021; Vernon and Blevins 2017). Other factors such as seasons also play a significant role in influencing the prevalence of *E. coli* in vegetables. For instance, contamination of cilantro and parsley with *E. coli* notably rises during the fall compared to levels observed in spring and winter (Lynch, Tauxe, and Hedberg 2009).

A large range of strains is seen in *E. coli* and these have the potential to cause significant harm (Zil‐e‐Huma Tareen et al. 2022). Drug‐resistant *E. coli* infections lengthen hospital stays, which puts economic pressure on the populace and healthcare systems both directly and indirectly (MacKinnon et al. 2020). It was discovered that plasmid‐directed mutations, which cause *E. coli* to become resistant to antibiotics, are extremely common in hospitals (Arbab et al. 2022). A previous study of *E. coli* antibiotic resistance revealed that it was approximately 70.00% resistant to streptomycin sulfisoxazole‐tetracycline. A drop in susceptibility to ampicillin, kanamycin, sulfasalazine, streptomycin, tetracycline, and ticarcillin was also observed (Tadesse et al. 2012). *E. coli* isolated from fresh salad vegetables showed resistance to ampicillin 30/145 (20.68%), tetracycline 29/145 (20.00%), and trimethoprim‐sulfamethoxazole 15/145 (10.35%). Multidrug‐resistance was present in *E. coli* isolates 20/145 (13.79%) (Habib et al. 2023). In a study on Bangladeshi vegetables, *E. coli* isolates displayed varying antibiotic resistance rates: 3/4 (75.00%) to erythromycin and cephalexin, 4/4 (100.00%) to gentamicin, ampicillin, and streptomycin, and 1/4 (25.00%) to chloramphenicol (Nigad Nipa et al. 2011). As previously mentioned, earlier studies found high *E. coli* levels in vegetables, exceeding BFSA limits (Ohiduzzaman Islam et al. 2022; Rahman and Noor 2012). Limited research has been conducted on antibiotic resistance in *E. coli* found in Bangladeshi vegetables. Factors such as water quality during washing, vendor handling practices, and environmental conditions, particularly seasonal variations, also influence the prevalence of *E. coli* in vegetables. Additionally, in Bangladesh, there is currently no research classifying *E. coli* isolated from vegetables based on its pathotypes. This research seeks to bridge existing knowledge gaps, providing insights into the classification of pathogenic *E. coli* , understanding their antibiotic resistance profiles, and assessing the impact of various factors such as water usage, vendor practices, and seasonal variations on vegetable contamination in Bangladesh.

## 2 Materials and Methods

### 2.1 Area and Sample Collection

Between June 2022 and March 2023, samples were taken from 14 local marketplaces inside the city of Dhaka. These areas are mentioned in Table S1.

In this study, 10 different types of fresh vegetables were collected during summer and winter seasons from different street vendors, grocery stores, and marketplaces from the aforementioned places in Dhaka city. These vegetables were chosen because they are frequently consumed raw and are typically not peeled. In our country, they are commonly eaten as salad vegetables. The 10 vegetable samples collected in this study are mentioned in Table S2.

Furthermore, the water used for washing vegetables and hand swabs from vendors were collected during the specified duration. Cross‐contamination of raw vegetables can occur while washing and handling the samples; hence, these samples were collected.

During the summer (June–October 2022) season, a total number of 513 samples (390 vegetables, 86 hand swabs, 37 water samples) and during the winter (December–March 2023) season, a total of 361 samples (250 vegetables, 86 hand swabs, 25 water samples) were collected. All of these samples were collected early in the morning. To avoid touch or cross‐contamination, sterile Ziplock bags (wiped with 70% ethanol) were used to collect salad vegetables. In the bazaars, a common tap was present. Vendors collected water from these taps in buckets, which they reused for washing vegetables by repeatedly submerging them. Samples of the water were collected directly from these buckets after the vegetables had been rinsed. The water samples were collected using high‐density plastic water sampling bottles. Before collection of these samples the bottles were vigorously washed and sterilized by autoclaving. From each water sample, 500 mL was collected. Sterilized test tubes and sterilized cotton swabs moistened with physiological saline were used to collect swabs from both hands of the vendors specifically finger tips. After labeling, all of the samples were transported in an ice box maintaining 4°C from the market to the microbiology laboratory without any delay.

### 2.2 Preparation of Samples

To prevent additional contamination, the vegetable samples were chopped inside a laminar airflow using a clean, disinfected knife. From each cut sample, 5 g was deposited in sterile, autoclaved conical flasks containing 25 mL of EC broth (HiMedia). This medium was chosen for enrichment purposes. This mixture was incubated in a shaker incubator at 120 rpm for a duration of 18–24 h at 37°C. As for the water samples and hand swabs, samples they were also enriched in EC broth and placed in a shaker incubator also at 120 rpm and 37°C for 18–24 h The above process was carried out following a modified guideline described by Waturangi, Hudiono, and Aliwarga (2019).

### 2.3 Enumeration of Total *E. coli* Count

The samples that were incubated overnight in EC broth were diluted 10‐fold using phosphate‐buffered saline (PBS), and 0.1 mL of each 10‐fold dilution was transferred and disseminated on MacConkey agar using the spread plate technique. The plates were then incubated at 37°C for 24 h. The colonies exhibiting lactose fermentation and displaying the required colony morphology were selected for total *E. coli* count. The overall bacterial count was represented as colony‐forming units per gram of vegetable, water, hand swab samples in its logarithmic value (log CFU/g).

### 2.4 Isolation and Identification

After overnight incubation, the suspected *E. coli* isolates were subcultured on Eosin Methylene Blue (EMB) (HiMedia, India) agar for further confirmation. Table S3 provides information on the selective media used and the corresponding colony morphology.

The isolated colonies were subcultured multiple times to measure colony homogeneity on EMB agar. Colony homogeneity was assessed by examining the colony morphology each time. The isolates were further identified by their colony morphology, Gram stain, and a series of biochemical tests. The biochemical tests included the triple sugar iron agar test, citrate test, the motility indole urease (MIU) test, methyl red test, Voges‐Proskauer test, catalase test, and oxidase test. Table S4 outlines the criteria for interpreting biochemical tests.

### 2.5 DNA Extraction and Amplification

The isolates were cultured in 1000 μL of brain heart infusion (BHI) broth and was incubated in a shaker incubator at 37°C overnight. The overnight culture was then centrifuged at 13,000–16,000× *g* for 2 min, and after that, the supernatant was removed. Then, the DNA was extracted by using the Wizard Genomic DNA Purification Kit from Promega Corp by following the manufacturer's instructions. To prepare a final solution of 25 μL of the PCR mixture for each isolate, the following components were combined: 5 μL of nuclease‐free water, 2 μL of forward primer, 2 μL of reverse primer, 12 μL of PCR Master Mix (EmeraldAmp), and 4 μL of template DNA. Molecular detection of pathogenic *E. coli* and their classification was performed using the Applied Biosystems 2720 Thermal Cycler thermocycler machine. The PCR products underwent gel electrophoresis for 50 min at 100 V and was examined on 1.5% agarose gel with 1X TAE buffer that was stained with ethidium bromide. Finally, the products were visualized under a UV transilluminator.

Specific genes were chosen to identify and classify the pathogenic strains of *E. coli* in the vegetable, water, and hand swab samples. To identify presumptive *E. coli* isolates, the 16S rRNA gene was targeted using ECO‐1 and ECO‐2 primers. For the identification of *E. coli* O157:H7 strains, the *eaeA*_*O157*_ gene was used. Detection of STEC involved the use of *stx1* and *stx2* genes, while EPEC was identified through *eaeA* genes. EIEC was detected using the *ipaH* gene, ETEC with the heat‐labile *eltB* toxin gene, and EAEC with the *CVD432* gene. The primer sequences, their PCR thermocycler conditions, and their amplicon sizes can be found in detail in Table S5.

### 2.6 Antibiotic Susceptibility Test

To determine the drug resistance pattern of *E. coli* , the Kirby–Bauer disc diffusion method was used to conduct antibiotic susceptibility tests. Turbidity was set to 0.5 McFarland by suspending colonies in 5 mL of 0.9% NaCl solution and then swabbed on Mueller Hinton Agar (MHA) (HiMedia). The zone diameters of the selected antimicrobial agents were interpreted following the Clinical Laboratory Standards Institute (CLSI) 2023 guidelines (Standards and Testing 2023). Single drug resistance is defined as resistance to a single antibiotic class, while multidrug resistance (MDR) is characterized by resistance to at least one agent in three or more antimicrobial categories. Extensively drug‐resistant (XDR) bacteria can be characterized using two main sets of criteria. The first set is based on the number of antimicrobials, classes, or subclasses to which a bacterium is resistant. The second set considers whether the bacterium is resistant to one or more key antimicrobial agents. These definitions follow the criteria established by the CLSI, European Committee on Antimicrobial Susceptibility Testing (EUCAST), and the United States Food and Drug Administration (FDA) (Magiorakos et al. 2012). In Table S6, a compilation of antibiotics used, along with their corresponding groups, disc potency, and interpretive criteria is given.

### 2.7 Statistical Analysis

To assess the significance of differences in *E. coli* prevalence rates across seasons, as well as the prevalence of specific pathotypes and antibiotic resistance patterns between seasons, a two‐way analysis of variance (ANOVA) was conducted using SPSS 29.0 for Windows (IBM) (Ohiduzzaman Islam et al. 2022). Estimated marginal means were performed to identify statistically significant differences among the combinations. A *p*‐value of < 0.05 was considered statistically significant for all tests.

### 2.8 Flowchart of the Methods Used in This Study

**FIGURE 1:**
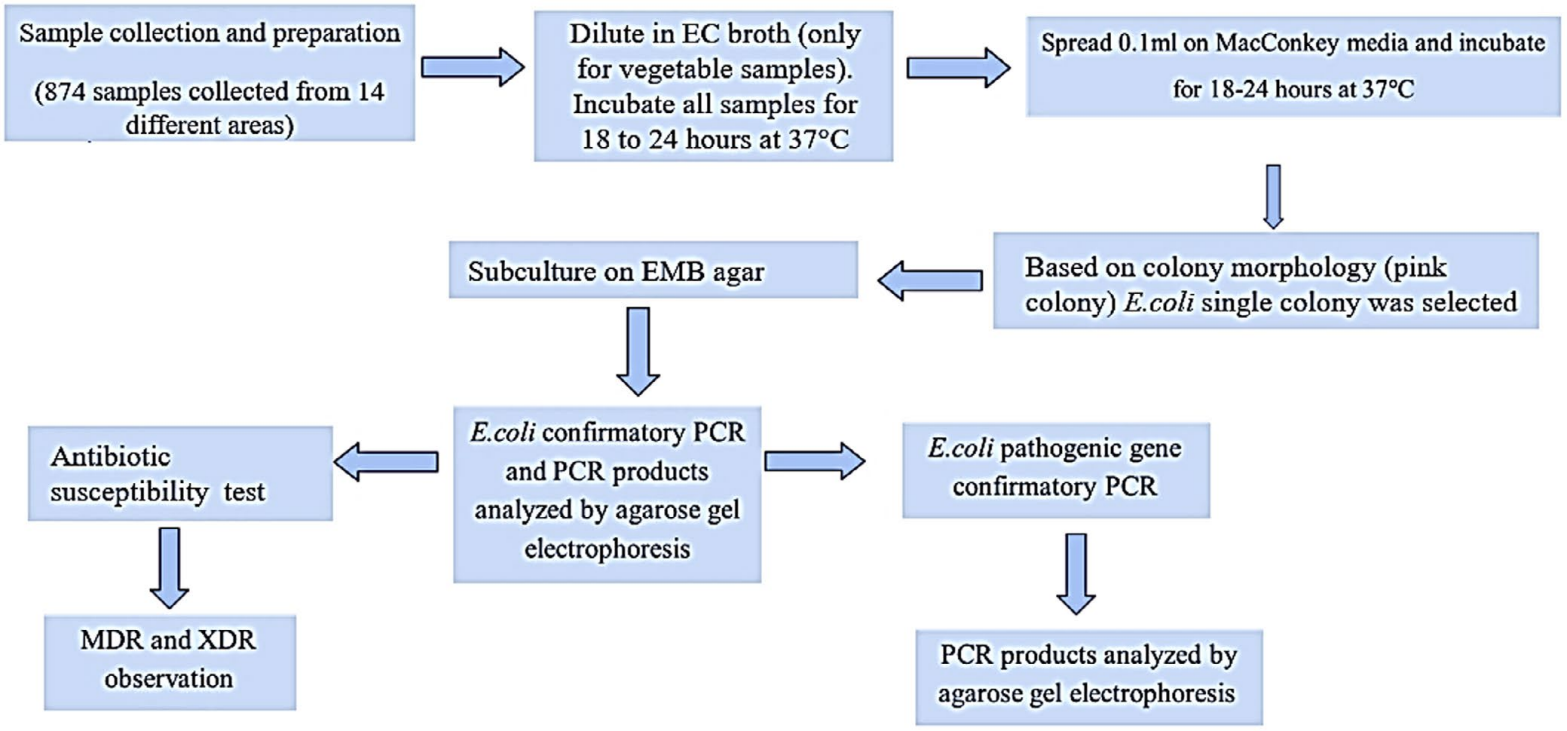
Flowchart detailing the methods used in this study.

The methods that were followed in this study are shown as a flowchart in Figure 1.

## 3 Results

In total, 874 samples were analyzed for the quantitative presence of *E. coli* during the winter and summer seasons in Dhaka city throughout the sampling period, out of which 782/874 (89.50%) samples were found positive. Of them, 622/640 (97.18%) were vegetables of different types, 99/172 (57.56%) were hand swabs of vendors, and 61/62 (98.39%) samples were of water that was used to wash vegetables. These isolates were identified through morphological, biochemical, and molecular examination.

High levels of *E. coli* were observed, especially in summer, where 490/513 (95.52%) samples were positive compared to 292/361 (80.87%) positive samples in winter. In summer, the prevalence of *E. coli* in water samples was 97.30% (*n* = 36/37), followed by vegetable samples 97.18% (*n* = 379/390) and hand swabs 87.21% (*n* = 75/86). Comparably, during winter, water samples had the highest percent prevalence 100.00% (*n* = 25/25), followed by vegetable samples 97.20% (*n* = 243/250) and hand swabs 27.91% (*n* = 24/86). To assess the significance of differences in *E. coli* prevalence rates across seasons, a two‐way analysis of variance (ANOVA) was performed. The results indicated that there was no statistically significant difference in the distribution of *E. coli* prevalence across seasons among the various samples. Information regarding this is described in detail in Table 1.

**TABLE 1:** Percentage of *Escherichia coli* isolated from vegetables, hand swabs, and vegetable washing water.

Summer		Winter		Overall	
Samples	Percentage of *E. coli n* (%)	Samples	Percentage of *E. coli n* (%)	Samples	Percentage of *E. coli* *n* (%)
Vegetables (*n* = 390)	379 (97.18%)	Vegetables (*n* = 250)	243 (97.20%)	Vegetables (*n* = 640)	622 (97.18%)
Hand swabs (*n* = 86)	75 (87.21%)	Hand swabs (*n* = 86)	24 (27.91%)	Hand swabs (*n* = 172)	99 (57.56%)
Vegetable washing water (*n* = 37)	36 (97.30%)	Vegetable Washing Water (*n* = 25)	25 (100.00%)	Vegetable Washing Water (*n* = 62)	61 (98.39%)
Total (*n* = 513)	490 (95.52%)	Total (*n* = 361)	292 (80.87%)	Total (*n* = 874)	782 (89.47%)

*Note:* Here, *n* indicates the number of positive isolates listed outside the parentheses, with the corresponding percentage given inside.

### 3.1 *E. coli* (Log CFU/g) Enumeration in Vegetables, Water, and Hand Swabs

After overnight incubation at 37°C in EC broth, 0.1 mL of each sample was spread on MacConkey agar to obtain the (log CFU/g) of *E. coli*. More *E. coli* was detected in summer than in winter from the same type of vegetables. The bacterial load of all sample types was within 4.95–5.94 log CFU/g, which exceeded the permissible limits of *E. coli* given by the BFSA and the ICMSF. Furthermore, the water samples exceeded the acceptable levels of *E. coli* given by the EC. Table 2 shows the range of bacterial load of the samples.

**TABLE 2:** Range of log CFU/g of *E. coli* isolates.

Sample name	Sample code	No. of samples (summer)	Sample containing *E. coli* (summer)	Range of bacterial load in log CFU/g (summer)	No. of samples (winter)	Sample containing *E. coli* (winter)	Range of bacterial load in log CFU/g (winter)
Lettuce	LET	41	41	5.14–5.75	24	24	5.32–5.85
Tomato	TOM	37	36	5.11–5.62	25	25	5.27–5.94
Capsicum	CAP	22	20	5.14–5.56	27	25	5.32–5.75
Cucumber	CU	34	31	5.14–5.79	22	21	5.27–5.79
Cabbage	CAB	29	27	5.32–5.79	25	25	5.17–5.63
Spring onion	SP	26	26	4.95–5.75	28	26	5.04–5.70
Carrot	CAR	40	39	5.11–5.82	37	35	5.44–5.67
Coriander	CORI	56	56	4.84–5.75	21	21	5.17–5.68
Mint	MINT	47	46	5.07–5.85	22	22	5.04–5.67
Green chili	GC	58	57	5.07–5.76	19	19	5.32–5.65
Hand swabs	HS	86	75	5.17–5.70	86	24	5.14–5.70
Water	W	37	36	4.95–5.62	25	25	5.04–5.63

*Note:* Here, log CFU/g represents the bacterial load of *E. coli* present in the samples.

### 3.2 Classification of the Pathogenic Strains of *E. coli* Isolates

Positive *E. coli* isolates collected in the summer season were tested for the *eaeA*_O157_ gene ( *E. coli* O157:H7 Strain), *stx1* and *stx2* genes (STEC), *eaeA* gene (EPEC), *ipaH* gene (EIEC), *eltB* gene (ETEC), and *CVD432* gene (EAEC) (Figures S1–S7). No sample tested positive for the *CVD432* gene, and therefore, EAEC was not present in our study in any of the seasons (Figure S8).

In *E. coli* isolates obtained from samples collected during the summer, the highest pathotype prevalence was observed for STEC (*stx2* gene) in hand swabs, with 9/36 (25.00%) of isolates testing positive, followed closely by STEC (*stx1* gene) at 8/36 (22.2%) and EIEC (*ipaH* gene) at 8/36 (22.22%). The most prevalent pathotype in vegetables was STEC (*stx1* gene), found in 73/379 (19.26%) samples, followed closely by STEC (*stx2* gene) in 69/379 (18.20%) samples. Other notable findings included EIEC (*ipaH* gene) in 59/379 (15.56%) of the samples and ETEC (*eltB* gene) in 44/379 (11.60%) of them. EPEC (*eaeA* gene) was present in 38/379 (10.02%) of the vegetables, while *E. coli* O157:H7 strain (*eaeA*_*O157*_ gene) was detected in 35/379 (9.23%) samples. In water samples, the lowest prevalence was recorded in multiple categories, including EPEC (*eaeA* gene) at 3/75 (4.00%), ETEC (*eltB* gene) at 3/75 (4.00%), and *E. coli* O157:H7 strain (*eaeA*_*O157*_ gene) at 5/75 (6.66%). This is described in detail in Table 3.

**TABLE 3:** *E. coli* isolates categorized based on virulent genes (summer samples).

Group	Targeted genes	Samples collected in summer		
		Number of isolates (%)		
		Vegetables (*n* = 379)	Water (*n* = 75)	Hand swabs (*n* = 36)
*E. coli* O157:H7 strain	*eaeA*_*O157*_	35 (9.23%)^a^	5 (6.66%)	5 (13.88%)^a^
STEC	*stx1*	73 (19.26%)^a^	10 (13.33%)^a^	8 (22.20%)^a^
	*stx2*	69 (18.20%)^a^	11 (14.66%)^a^	9 (25.00%)^a^
EPEC	*eaeA*	38 (10.02%)^a^	3 (4.00%)	4 (11.11%)^a^
EIEC	*ipaH*	59 (15.56%)^a^	9 (12.00%)^a^	8 (22.22%)^a^
ETEC	*eltB*	44 (11.60%)^a^	3 (4.00%)	5 (13.88%)^a^
EAEC	*CVD432*	0 (0.00%)	0 (0.00%)	0 (0.00%)

*Note:* Here, *n* indicates the number of positive isolates listed outside the parentheses, with the corresponding percentage given inside.

^a^ Indicates the combinations that are statistically significant.

In *E. coli* isolates obtained from samples collected during the winter, it was observed that the highest prevalence of *E. coli* O157:H7 strains and *E. coli* pathotypes was in vegetable samples, while the lowest was found in the hand swabs of vegetable vendors. *E. coli* O157:H7 strain (*eaeA*_*O157*_ gene) was detected in 18/243 (6.21%) vegetable samples, which was higher than in water (3/25, 2.91%) and hand swabs (2/24, 2.30%). STEC showed the most significant difference in prevalence, especially in vegetables. The *stx1* gene was found in 27/243 (9.31%) vegetable samples compared to 9/25 (8.73%) water samples and 5/24 (5.75%) hand swabs. Similarly, the *stx2* gene was detected in 30/243 (10.34%) vegetable samples, which is markedly higher than in water (8/25, 7.77%) and hand swabs (4/24, 4.60%). EPEC carrying the *eaeA* gene was found in 21/243 (7.24%) of vegetables, again higher than in water (4/25, 3.89%) and hand swabs (2/24, 2.30%). EIEC was present in 26/243 (8.97%) vegetable samples compared to 6/25 (5.82%) water samples and 4/24 (4.60%) hand swabs. ETEC was detected in 20/243 (6.90%) of vegetable samples, higher than in water (4/25, 3.89%) and hand swabs (3/24, 3.45%). This is described in detail in Table 4.

**TABLE 4:** *E. coli* isolates categorized based on virulent genes (winter samples).

Group	Targeted genes	Samples collected in winter		
		Number of isolates (%)		
		Vegetables (*n* = 243)	Water (*n* = 25)	Hand swabs (*n* = 24)
*E. coli* O157:H7 strain	*eaeA*_*O157*_	18 (6.21%)^a^	3 (2.91%)	2 (2.30%)^a^
STEC	*stx1*	27 (9.31%)^a^	9 (8.73%)^a^	5 (5.75%)^a^
	*stx2*	30 (10.34%)^a^	8 (7.77%)^a^	4 (4.60%)^a^
EPEC	*eaeA*	21 (7.24%)^a^	4 (3.89%)	2 (2.30%)^a^
EIEC	*ipaH*	26 (8.97%)^a^	6 (5.82%)^a^	4 (4.60%)^a^
ETEC	*eltB*	20 (6.90%)^a^	4 (3.89%)	3 (3.45%)^a^
EAEC	*CVD432*	0 (0.00%)	0 (0.00%)	0 (0.00%)

*Note:* Here, *n* indicates the number of positive isolates listed outside the parentheses, with the corresponding percentage given inside.

^a^ Indicates the combinations that are statistically significant.

A two‐way ANOVA revealed that seasons significantly affect *E. coli* pathotype prevalence in vegetable, water, and hand swab samples. For both vegetable and hand swab samples, *E. coli* pathotype prevalence was consistently higher in summer for all genes except *CVD432*. In water samples, the *ipaH* gene exhibited a statistically significant difference between summer and winter, with higher prevalence in summer. Similarly, the *stx1* and *stx2* genes also demonstrated statistically significant differences, further supporting the notion of meaningful seasonal variation. In contrast, the *eaeA*, *eaeA*_*O157*_, *eltB*, and *CVD432* genes showed no statistically significant differences between the seasons.

In the vegetable samples, spring onions showed the highest prevalence of the *eaeA*_*O157*_ gene, with 5/26 samples (19.23%) testing positive. The *stx1* gene (STEC) was found in 8/26 samples (30.76%). Capsicum exhibited the highest rates for several genes: *stx2* (STEC) in 8/20 samples (40.00%), *eaeA* (EPEC) in 4 /20 samples (20.00%), *ipaH* (EIEC) in 7/20 samples (35.00%), and *eltB* (ETEC) in 4/20 samples (20.00%) (Table S7). During the winter, coriander had the highest rate of *eaeA*_O157_ (14.28%, 3/21 samples). Cucumber samples contained the most *stx1* gene (19.04%, 4/21 samples), while tomato and capsicum showed the highest levels of *stx2* (16.00%, 4/25 samples). Carrot samples had the highest prevalence of the *eaeA* gene (11.42%, 4/35 samples). Coriander showed the most *ipaH* gene (14.28%, 3/21 samples), and tomato had the highest prevalence of the *eltB* gene (16.00%, 4/25 samples) (Table S8). A two‐way ANOVA revealed no statistically significant differences in the prevalence of the *eaeA*_*O157*_, *stx2*, *eaeA*, *ipaH*, and *eltB* genes between summer and winter across the different vegetable samples. While some vegetables exhibited higher mean levels in summer, these differences did not reach statistical significance. However, a statistically significant seasonal difference was found for the *stx1* gene, where all vegetable samples (cabbage, capsicum, carrot, coriander, cucumber, green chili, lettuce, mint, spring onion, and tomato) displayed significant differences in *E. coli* pathotype prevalence between summer and winter, with higher levels observed in summer for each.

### 3.3 Antibiotic Susceptibility Pattern of *E. coli* Isolates

In this study, a total of 1206 isolates (summer 726 isolates and winter 480 isolates) have been selected for antibiotic susceptibility testing to observe the antibiotic susceptibility pattern of *E. coli* .

#### 3.3.1 Summer Sample Antibiotic Susceptibility Pattern of the Isolates

The antimicrobial resistance profile indicates that *E. coli* isolates detected in vegetables, water samples, and hand swabs were highly resistant to ampicillin 471/489 (96.32%), 126/127 (99.21%), and 108/110 (98.18%), respectively, and erythromycin 416/489 (85.07%), 115/127 (90.55%), and 85/110 (77.27%), respectively. The results of the antibiotic susceptibility pattern of isolates detected in summer are shown in Table 5.

**TABLE 5:** Summer sample antibiotic susceptibility pattern of *E. coli* isolates.

Antibiotic name	Vegetable			Water			Hand swabs		
	Number of isolates (%) (*n* = 489)			Number of isolates (%) (*n* = 127)			Number of isolates (%) (*n* = 110)		
	Sensitive	Intermediate	Resistant	Sensitive	Intermediate	Resistant	Sensitive	Intermediate	Resistant
Amikacin	395 (80.78%)	27 (5.52%)	67 (13.70%)^a^	107 (84.25%)	1 (0.79%)	19 (14.96%)	95 (86.36%)	4 (3.64%)	11 (10.00%)
Gentamicin	462 (94.48%)	15 (3.07%)	12 (2.45%)	127 (100.00%)	0 (0.00%)	0 (0.00%)	101 (91.81%)	4 (3.64%)	5 (4.55%)
Kanamycin	288 (58.90%)	118 (24.34%)	83 (16.97%)^a^	82 (64.57%)	23 (18.11%)	22 (17.32%)	80 (72.73%)	24 (21.81%)	6 (5.45%)
Streptomycin	393 (80.00%)	78 (15.95%)	18 (3.68%) ^†^	114 (90.00%)	12 (9.49%)	1 (0.79%)	103 (93.63%)	7 (6.36%)	0 (0.00%)
Imipenem	473 (96.72%)	8 (1.63%)	8 (1.64%)	126 (99.21%)	0 (0.00%)	1 (0.79%)	107 (97.27%)	1 (0.91%)	2 (1.82%)
Meropenem	427 (87.32%)	58 (11.86%)	4 (0.82%)	97 (76.38%)	26 (20.47%)	4 (3.15%)	94 (85.45%)	14 (12.72%)	2 (1.82%)
Ampicillin	10 (2.04%)	8 (1.64%)	471 (96.32%)^a^	0 (0.00%)	1 (0.79%)	126 (99.21%)	2 (1.82%)	0 (0.00%)	108 (98.18%)
Ciprofloxacin	423 (86.50%)	30 (6.13%)	36 (7.36%)^a^	87 (68.50%)	24 (18.90%)	16 (12.60%)	84 (76.36%)	9 (8.18%)	17 (15.45%)
Erythromycin	0 (0.00%)	73 (14.92%)	416 (85.07%)^a^	0 (0.00%)	12 (9.49%)	115 (90.55%)	0 (0.00%)	25 (22.72%)	85 (77.27%)
Chloramphenicol	400 (81.80%)	39 (7.98%)	50 (10.22%)^a^	115 (90.55%)	9 (7.08%)	3 (2.36%)	101 (91.81%)	7 (6.36%)	2 (1.82%)
Tetracycline	429 (87.73%)	45 (9.20%)	15 (3.06%)^a^	110 (86.61%)	13 (10.24%)	4 (3.15%)	99 (90.00%)	6 (5.45%)	5 (4.45%)
Colistin	67 (13.70%)	309 (63.19%)	113 (23.11%)^a^	34 (26.77%)	71 (55.91%)	22 (17.32%)	27 (24.55%)	66 (60.00%)	17 (15.45%)

*Note:* Here, *n* indicates the number of positive isolates listed outside the parentheses, with the corresponding percentage given inside.

^a^ Indicates the combinations that are statistically significant.

#### 3.3.2 Winter Sample Antibiotic Susceptibility Pattern of the Isolates

Similar to the summer samples, *E. coli* isolates detected in vegetables, water samples, and hand swabs were highly resistant to ampicillin 285/290 (98.28%), 102/103 (99.03%), and 85/87 (97.70%), respectively, and erythromycin 267/290 (92.07%), 98/103 (95.15%), and 68/87 (78.16%), respectively. The results of the antibiotic susceptibility pattern of isolates detected in winter are shown in Table 6.

**TABLE 6:** Winter samples’ antibiotic susceptibility pattern of *E. coli* isolates.

Antibiotic name	Vegetable			Water			Hand swabs		
	Number of isolates (%) (*n* = 290)			Number of isolates (%) (*n* = 103)			Number of isolates (%) (*n* = 87)		
	Sensitive	Intermediate	Resistant	Sensitive	Intermediate	Resistant	Sensitive	Intermediate	Resistant
Amikacin	258 (88.97%)	14 (4.83%)	18 (20.69%)^a^	88 (85.44%)	7 (6.80%)	8 (7.76%)	78 (89.65%)	3 (3.44%)	6 (6.89%)
Gentamicin	267 (92.07%)	11 (3.80%)	12 (4.14%)	102 (99.03%)	1 (0.97%)	0 (0.00%)	79 (90.80%)	4 (4.60%)	4 (4.60%)
Kanamycin	163 (56.21%)	84 (28.97%)	43 (14.83%)^a^	69 (66.99%)	16 (15.53%)	18 (17.47%)	69 (79.31%)	14 (16.09%)	4 (4.60%)
Streptomycin	186 (64.00%)	48 (16.55%)	56 (19.31%)^a^	93 (90.00%)	9 (8.74%)	1 (0.97%)	81 (93.00%)	6 (6.90%)	0 (0.00%)
Imipenem	218 (75.17%)	65 (22.41%)	7 (2.41%)	75 (72.82%)	17 (16.50%)	11 (10.68%)	66 (75.86%)	16 (18.39%)	5 (5.75%)
Meropenem	265 (91.38%)	25 (8.62%)	0 (0.00%)	80 (77.67%)	19 (18.47%)	4 (3.88%)	76 (87.36%)	10 (11.49%)	1 (1.15%)
Ampicillin	1 (0.34%)	4 (1.38%)	285 (98.28%)^a^	0 (0.00%)	1 (0.97%)	102 (99.03%)	2 (2.30%)	0 (0.00%)	85 (97.70%)
Ciprofloxacin	251 (86.55%)	16 (5.51%)	23 (7.93%)^a^	75 (77.82%)	17 (16.50%)	11 (10.68%)	71 (81.70%)	6 (6.70%)	10 (11.49%)
Erythromycin	0 (0.00%)	23 (7.93%)	267 (92.07%)^a^	0 (0.00%)	5 (4.85%)	98 (95.15%)	0 (0.00%)	19 (21.84%)	68 (78.16%)
Chloramphenicol	224 (77.24%)	19 (6.55%)	47 (16.20%)^a^	93 (90.29%)	7 (6.79%)	3 (2.91%)	78 (89.65%)	7 (8.05%)	2 (2.30%)
Tetracycline	269 (92.76%)	3 (1.03%)	18 (6.20%)^a^	90 (87.38%)	10 (9.70%)	3 (2.91%)	78 (89.66%)	4 (4.60%)	5 (5.75%)
Colistin	25 (8.62%)	197 (67.93%)	68 (23.45%)^a^	29 (28.15%)	60 (58.25%)	14 (13.59%)	22 (25.29%)	57 (65.52%)	8 (9.20%)

*Note:* Here, *n* indicates the number of positive isolates listed outside the parentheses, with the corresponding percentage given inside.

^a^ Indicates the combinations that are statistically significant.

A two‐way ANOVA revealed significant seasonal differences in resistance levels for vegetable samples against the tested antibiotics, while no significant differences were observed for water and hand swab samples. For vegetable samples, statistically significant seasonal differences in resistance were noted for amikacin, ampicillin, chloramphenicol, ciprofloxacin, colistin, erythromycin, kanamycin, streptomycin, and tetracycline. However, in hand swab samples, some antibiotics, such as amikacin and ciprofloxacin, exhibited higher resistance means in summer compared to winter, although these differences were not statistically significant.

**FIGURE 2:**
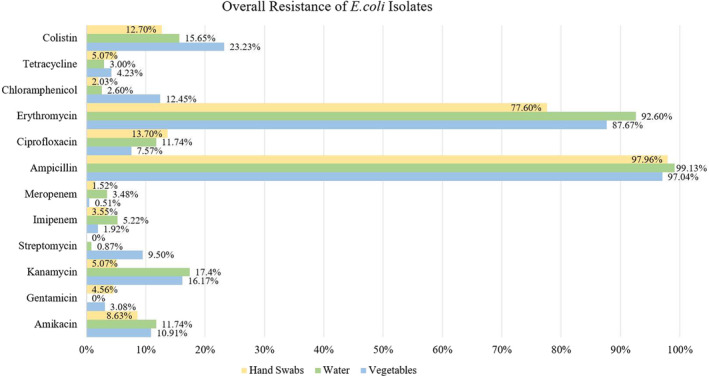
Overall resistance of *E. coli* isolates from summer and winter seasons.

Overall, *E. coli* isolates showed the highest resistance to ampicillin antibiotics across all samples, followed by erythromycin. *E. coli* isolates from all samples exhibited the lowest resistance to gentamicin, streptomycin, imipenem, and meropenem antibiotics. This is detailed in Figure 2.

During the summer, the highest percentage of MDR was observed in spring onion samples 30/53 (56.60%). XDR isolates were observed in mint 1/46 (2.17%) and cabbage 1/51 (1.96%) samples. During winter, the highest percentage of MDR was observed in carrot samples 23/32 (71.87%). XDR isolates were also observed in carrot samples 2/32 (6.25%). Table 7 describes the counts and percentages of MDR and XDR isolates of both seasons. Table S9 presents the area‐wise distribution of vegetable samples, water samples, and hand swabs collected from Dhaka City in the summer, along with the corresponding counts of MDR and XDR isolates. Table S10 presents the area‐wise distribution of vegetable samples, water samples, and hand swabs collected from Dhaka City in winter, along with the corresponding counts of MDR and XDR isolates.

**TABLE 7:** Percentages of multidrug‐resistant and extensively drug‐resistant isolates for both seasons.

Sample name	Summer			Winter		
	Total isolates	No. of MDR isolates (%)	No. of XDR isolates (%)	Total isolates	No. of MDR isolates (%)	No. of XDR isolates (%)
Lettuce	53	22 (41.50%)	0 (0.00%)	32	10 (31.25%)	0 (0.00%)
Tomato	47	21 (44.68%)	0 (0.00%)	40	21 (52.50%)	0 (0.00%)
Capsicum	43	18 (41.86%)	0 (0.00%)	31	21 (67.74%)	0 (0.00%)
Cucumber	51	15 (29.41%)	0 (0.00%)	22	11 (50.00%)	0 (0.00%)
Cabbage	51	26 (50.98%)	1 (1.96%)	28	11 (39.28%)	0 (0.00%)
Spring onion	53	30 (56.60%)	0 (0.00%)	23	14 (60.86%)	0 (0.00%)
Carrot	43	21 (48.83%)	0 (0.00%)	32	23 (71.87%)	2 (6.25%)
Coriander	50	24 (48.00%)	0 (0.00%)	28	15 (53.57%)	0 (0.00%)
Mint	46	19 (41.30%)	1 (2.17%)	27	15 (55.55%)	0 (0.00%)
Green chili	52	22 (42.30%)	0 (0.00%)	27	16 (59.25%)	0 (0.00%)
Water	127	60 (47.24%)	0 (0.00%)	103	46 (44.66%)	0 (0.00%)
Hand swab	110	41 (37.27%)	0 (0.00%)	87	28 (32.18%)	0 (0.00%)
Total	726	319 (43.93%)	2 (0.27%)	480	231 (48.12%)	2 (0.41%)

*Note:* The number of positive isolates is listed outside the parentheses, with the corresponding percentage given inside.

## 4 Discussion

Globally, the agricultural sector has witnessed a substantial surge in vegetable production, experiencing a remarkable 94.00% increase from 1980 to 2004, primarily driven by population growth (*Food Net Surveillance Report for 2004* 2006). This upward trajectory in production has continued over the past 18 years (2000 to 2018), with the overall output rising from 682.43 to 1088.9 million metric tons, although a slight decline was observed between 2017 and 2018. This increase in vegetable production is notably distributed among developing countries, with nations such as India, Iran, Vietnam, Nigeria, Egypt, and Mexico emerging as prominent contributors, ranking among the top 10 global vegetable producers (FAO 2017). As a result of this expanded production, there has been a simultaneous acceleration in the consumption of vegetables across different regions. Asian countries have emerged as the highest consumers, followed by Europe, Northern America, Oceania, and Africa (Balali et al. 2020). Moreover, the consumption rate of vegetables, particularly in developing countries, demonstrated a significant uptrend, experiencing a notable 69.00% increase from 1961 to 2013 (FAO 2017). Over the past three decades, there has been a notable 25.00% increase in the average per‐person consumption of fresh produce in the United States (Harrington 2009). A diet abundant in fruits and vegetables has demonstrated protective effects against various cancers and chronic ailments, such as coronary heart disease (Goodburn and Wallace 2013). Despite the numerous advantages that vegetable consumption offers for our well‐being, it can have adverse effects on our health if proper food safety practices are not diligently followed. This is because vegetables provide various opportunities for the adherence and invasion of harmful microbes or parasites throughout their cultivation, harvesting, processing, and marketing stages. This situation has led to a rising incidence of foodborne outbreaks attributed to bacterial contamination of these products (Takeuchi, Hassan, and Frank 2001). In the United States, data from 1990 to 2005, as reported by the Food Safety Project, revealed a minimum of 713 produce‐related outbreaks associated with foodborne diseases, with 12.00% involving fresh fruits and vegetables (Goodburn and Wallace 2013; Hubbard 2004). The most recent *E. coli* outbreak linked to vegetables in the USA occurred in 2021. The CDC reported outbreaks of *E. coli* O157:H7 associated with baby spinach and packaged salads, resulting in one fatality ( *E. coli Outbreak Linked to Baby Spinach* 2021; *E. coli Outbreak Linked to Packaged Salads* 2021). Furthermore, the ACMSF reported 531 cases of illness, including one fatality, linked to various foodborne diseases in the United Kingdom between 2008 and 2010 (O'Brien 2011).

In Bangladesh, the revenue in fresh vegetables market amounts to US$20.45 billion in 2024, with an anticipated annual growth rate of 9.92% (CAGR 2024–2028) (*Vegetables—Bangladesh. Statista Market Forecast* 2024). These figures highlight a robust and expanding market. In Bangladesh, raw vegetables play a significant role in the dietary habits of the population. On average, each person consumes 23 g of leafy vegetables, 89 g of non‐leafy vegetables, and 14 g of fruit daily, amounting to a total of 126 g of fruits and vegetables per day. A study conducted in rural areas revealed that 39.00% of respondents consumed vegetables 3 to 6 times per day, 34.00% ate them 2 to 3 times per day, 14.00% had them once a day, while 13.00% consumed vegetables only 1 to 2 times per month (Monoarul Haque et al. 2014). Another study conducted in various regions of Bangladesh found that respondents consumed vegetables almost daily, averaging 6.95 ± 0.27 days per week (Rahman et al. 2021). From a food consumption and dietary assessment survey conducted by the Food and Agriculture Organization (FAO) in collaboration with the Institute of Nutrition and Food Science, Dhaka University (INFS) between April and October 2004, it was observed that raw vegetables were a prevalent component of the diet, with 80.00% of project households including fresh carrots, all households consuming fresh cucumbers, 76.00% incorporating raw onions, 74.00% including fresh radishes, 98.00% consuming uncooked tomatoes, and 12.00% including fresh green chilies. The survey also revealed that the average household consumption of vegetables stood at 48 kg in winter and 40 kg in summer. Per capita consumption was measured at 53.30 g in winter and 43 g in summer, indicating seasonal variations in vegetable consumption patterns (Bhattacharjee, Kumar Saha, and Nandi 2007). In Bangladesh, approximately 30 million individuals are reported to be affected by *E. coli* ‐related foodborne diseases (Afzalur Rahman et al. 2018), and the latest *E. coli* outbreak occurred in 2021 in the Barisal division, resulting in eight fatalities and the hospitalization of thousands (Dhali 2021).

Previous studies conducted in Bangladesh have revealed concerning findings regarding *E. coli* contamination in vegetables. A Bangladeshi study focusing on raw salad vegetables found a lower prevalence of *E. coli* , standing at 13/266 (4.89%) (Nigad Nipa et al. 2011). Other studies showed that *E. coli* contamination in salad vegetables had a substantial bacterial load ranging from 10^4^ to 10^8^ CFU/g, (Ohiduzzaman Islam et al. 2022; Rahman and Noor 2012). Factors such as water quality during washing, vendor handling practices, and environmental conditions, notably seasonal variations, have been identified as influencing the prevalence of *E. coli* in vegetables. Despite the significant consumption of vegetables in Bangladesh, there is limited research on antibiotic resistance in *E. coli* derived from these vegetables, an issue that urgently needs to be addressed given the global concern over antibiotic resistance and its implications for public health.

Between June 2022 and March 2023, samples were taken from 14 local marketplaces (Table S1) inside Dhaka. In this study, 10 different types of fresh vegetables (Table S2) were collected during summer and winter seasons from different street vendors, grocery stores, and marketplaces from different places in Dhaka city. These vegetables were chosen because they are frequently consumed raw and are typically not peeled.

Furthermore, the water used for washing vegetables and hand swabs from vendors were collected during the specified duration. Cross‐contamination of raw vegetables can occur during the washing and handling process. Due to this, samples of the water used to wash the vegetables and hand swabs from vendors were collected. During the summer (June–October 2022) season, a total number of 513 samples (390 vegetables, 86 hand swabs, 37 water samples) and during the winter (December–March 2023) season, a total of 361 samples (250 vegetables, 86 hand swabs, 25 water samples) were collected.

Elevated levels of *E. coli* were noted, particularly during the summer, where 490/513 (95.52%) samples tested positive as opposed to 292/361 (80.87%) positive samples in winter. It is noteworthy that the higher count in summer may be attributed to a lower number of samples collected during the winter season. In summer, the prevalence of *E. coli* in water samples was 97.30% (36/37), followed by vegetable samples at 97.18% (379/390) and hand swabs at 87.21% (75/86). Similarly, in winter, water samples exhibited the highest percentage prevalence at 100.00% (25/25), followed by vegetable samples at 97.2% (243/250) and hand swabs at 27.91% (24/86). A two‐way analysis of variance (ANOVA) was used to check if *E. coli* prevalence rates varied by season. The results showed no significant differences in *E. coli* prevalence across seasons among the different samples. However, a prior study conducted in Vietnam reported a lower prevalence of *E. coli* at 95/200 (47.50%) in water samples used to moisten vegetables, contrasting with our findings (Tram and Dalsgaard 2014).

As previously mentioned according to the BFSA, the presence of *E. coli* O157:H7 or Vero or STEC in 25 g of cut or minimally processed and packed (non‐thermally processed) vegetables should be entirely absent. For ready‐to‐eat packaged fresh vegetables, the permissible level of *E. coli* is 10^1^–10^2^ CFU/g (Sarker 2023). Additionally, according to the ICMSF, acceptable *E. coli* levels in frozen fruits and vegetables (pH > 4.5) and dried vegetables are 10^2^–10^3^ CFU/g (Stewart 1987). The bacterial load of all the samples was recorded in log CFU/g. Notably, all the vegetable samples exceeded the permissible limits (Table 2). The highest bacterial load was detected in mint samples (5.85 log CFU/g) collected in the summer, while the highest bacterial load in winter was found in tomato samples (5.94 log CFU/g). In a previous study conducted in Bangladesh, it was found that lettuce samples had the highest bacterial load at 5 × 10^8^ CFU/g. Cucumbers, carrots, and tomatoes were also examined, revealing their respective highest bacterial loads of 4.42 × 10^7^, 3.48 × 10^7^, and 1.23 × 10^8^ CFU/g (Rahman and Noor 2012). In another study conducted in Bangladesh, it was found that carrot samples had the highest level of *E. coli* contamination (7.58 ± 1.55 log CFU/g). Other vegetable samples tested were tomatoes, cucumbers coriander leaves, and green chilies, and their highest level of contamination was 7.36 ± 0.64, 6.59 ± 0.33, 6.91 ± 0.47, and 6.61 ± 0.98 log CFU/g, respectively (Ohiduzzaman Islam et al. 2022). The contamination levels observed in these studies were significantly higher than those identified in our research. In a separate study in Bangladesh in 2019, it was observed that *E. coli* levels in lettuce ranged from 1.69 to 3.75 log CFU/g, the count in tomatoes ranged from 1.69 to 2.42 log CFU/g, cucumber *E. coli* count ranged from 0.67 to 3.81 log CFU/g, and *E. coli* in coriander leaves ranged from 3.85 to 6.34 log CFU/g (Ahmed et al. 2019). Previous research has highlighted varying levels of *E. coli* contamination in raw vegetables across different regions. In Cambodia, lettuce, cucumber, and tomato showed contamination levels of 2.71 ± 0.97, 2.28 ± 0.95, and 2.18 ± 0.16 log CFU/g, respectively (Schwan et al. 2022). In Kenya, vegetable salads consisting of tomatoes, onions, and chili peppers served in canteens at Egerton University exhibited an average *E. coli* O157: H7 count of 3.047 log CFU/g (Osafo et al. 2022). Similarly, in the UAE, a study found that a significant portion 106 /400 (26.50%) of fresh salad vegetables, particularly arugula and spinach from retail outlets, contained unsatisfactory levels of *E. coli* counts (> 100 CFU/g) (Habib et al. 2023). Past studies have suggested that leafy vegetables, such as arugula and spinach, are more prone to surface attachment, thereby enhancing the survival likelihood of certain bacterial groups (Hölzel, Tetens, and Schwaiger 2018; Mohammad, Do Prado, and Sirsat 2022). The differences observed between our study and others can be attributed to various factors such as differences in methodology, geographical locations, climate conditions, and production practices across the study settings. These disparities emphasize the need for careful consideration of context when interpreting findings on *E. coli* contamination in vegetables. When it comes to selling vegetables, both supermarkets and local markets must adhere to necessary precautions regarding proper handling and hygienic practices. This is particularly crucial because salad vegetables are often stored at room temperature for extended periods during the sales process (Ohiduzzaman Islam et al. 2022). *E. coli* contamination serves as an indicator of fecal contamination, suggesting an increased risk for the presence of zoonotic pathogens such as *Salmonella, Campylobacter*, and verotoxin‐producing *E. coli* . These pathogens are typically excreted in feces by animals (Jensen et al. 2013). Therefore, maintaining rigorous hygiene standards throughout the production and distribution chain is essential to mitigate the risk of foodborne illnesses associated with contaminated vegetables.

The EC permits the use of clean water with an *E. coli* level of 100 CFU/100 mL for post‐harvest cooling, transportation of non‐ready‐to‐eat fresh fruits and vegetables, and initial washing of ready‐to‐eat produce (“Safety and Quality of Water Used in Food Production and Processing” 2019). The *E. coli* levels in all water samples during both summer (5.17–5.70 log CFU/g) and winter (5.14–5.70 log CFU/g) exceeded the permissible limits. Water utilized in post‐harvest handling and processing operations tends to accumulate organic matter and microorganisms that originate from soil, plant exudates, and debris (Koutsoumanis et al. 2020). A prior study conducted in Hanoi revealed that out of 200 water samples used to keep vegetables moist, 95/200 (47.50%) of them tested positive for *E. coli* , with a median concentration of 636 CFU/mL (Tram and Dalsgaard 2014).

In our study, it was observed that most of the hand swab samples 91/172 (57.56%) were positive for a high level of *E. coli* (4.95–5.62 log CFU/cm^2^). In a previous study, it was observed that the level of *E. coli* in hand swabs of food handlers was 0.2 ± 0.4 log CFU/cm^2^ (Tan, Lee, and Mahyudin 2014). Another study in Qatar showed that the level of *E. coli* in hand swabs of food handlers was 5.08–5.54 log CFU/cm^2^, which agrees with our findings (El‐Nemr et al. 2019). Multiple studies indicate that food workers frequently practice unsafe food handling, resulting in the microbial contamination of ready‐to‐eat foods. This commonly arises from food handlers being asymptomatic carriers of pathogenic microorganisms or having insufficient personal hygiene. Reducing the risk of contamination by food workers entails implementing thorough handwashing protocols and improving personal hygiene practices (Faour‐Klingbeil et al. 2016). In accordance with the Food Safety Modernization Act (FSMA) for fresh products, food handlers should receive training on the correct use of sanitizing agents and on the principles of food hygiene and safety (*Food and Drugs* 2015).

Positive *E. coli* isolates were assessed for various genes representing different pathotypes. The *CVD432* gene, indicative of EAEC, was not detected in our study. During the summer, hand swabs had the highest presence of *E. coli* O157:H7, while water used for washing vegetables had the lowest (Table 3). Among vegetables, spring onions had the highest *E. coli* O157:H7 rate 5/26 (19.23%), with the *stx1* gene (STEC) found in 8/26 (30.76%) of samples. Capsicum showed the highest rates for the *stx2* gene (STEC) at 8/20 (40.00%), the *eaeA* gene (EPEC) at 4/20 (20.00%), the *ipaH* gene (EIEC) at 7/20 (35.00%), and the *eltB* gene (ETEC) at 4/20 (20.00%) (Table S7). These results could be influenced by the lower number of capsicum samples. In winter, the highest concentration of *eaeA*_*O157*_ was found in vegetable samples, while the lowest was in hand swabs (Table 4). Coriander had the highest rate of *eaeA*_*O157*_ *E. coli* O157:H7 3/21 (14.28%). Cucumber samples had the most *stx1* gene (STEC) at 4/21 (19.04%), while tomato and capsicum showed the highest levels of *stx2* gene (STEC) at 4/25 (16.00%). Carrot samples had the most *eaeA* gene (EPEC) at 4/35 (11.42%). Coriander had the most *ipaH* gene (EIEC) at 3/21 (14.28%), and tomato had the highest *eltB* gene (ETEC) at 4/25 (16.00%) (Table S8). In the case of both summer and winter vegetable samples, *E. coli* O157:H7 strains identified were not positive for STEC. A two‐way ANOVA revealed no statistically significant differences in *eaeA*_*O157*_, *stx2, eaeA, ipaH*, and *eltB* gene prevalence between summer and winter across the different vegetable samples. However, there was a statistically significant seasonal difference for the *stx1* gene, with consistently higher prevalence in summer across all vegetable types.

Several previous studies have examined the prevalence of different pathotypes of *E. coli* in various fruits and vegetables, revealing significant findings across different regions. In Jakarta, for instance, a study found that 55/76 (72.37%) of samples tested positive for EAEC, with isolates detected in tomato, starfruit, guava, cucumber, and cabbage. Additionally, 12/76 (15.79%) of samples were positive for EPEC exclusively found in tomatoes, while 9/76 (11.84%) tested positive for ETEC, isolated solely from cabbage samples in the South of Jakarta (Waturangi, Hudiono, and Aliwarga 2019). Similarly, in the Czech Republic, the *eaeA* gene associated with EPEC was identified in 3/27 (11.10%) of isolates, recovered from asparagus, chickpea sprouts, and spring onions (Skočková et al. 2013). Meanwhile, in another study conducted in Mexico, EPEC, ETEC, and STEC were each isolated from 3/220 (1.40%) of samples, with only STEC strains containing the *stx1* locus being identified (Bautista‐De León et al. 2013). Another Mexican study found STEC, EIEC, EPEC, and ETEC in different types of tomatoes, with ETEC present in 6/100 (6.00%) of saladette tomatoes and 5/100 (5.00%) of red round tomatoes, while EPEC was found in 2/100 (2.00%) of saladette samples and 3/100 (3.00%) of red round samples. EIEC was detected in 1/100 (1.00%) of saladette samples (Gómez‐Aldapa et al. 2013). In Malaysia, *E. coli* O157 was found in 3/30 (10.00%) of bean samples and 1/30 (3.30%) of white radish samples (Chang et al. 2013). The presence of different *E. coli* pathotypes in vegetables can be attributed to various factors. *E. coli* O157:H7, for instance, has demonstrated its ability to colonize and internalize primarily in live spinach and lettuce plants (Berger et al. 2010; Wright et al. 2013). It exhibits persistence by being bound or entrapped in different parts of the plant, such as leaves, sprouts, and fruit, even after thorough washing or disinfection (Beuchat 1999; Franz et al. 2007; Jeter and Matthysse 2005; Solomon, Yaron, and Matthews 2002; Taormina and Beuchat 1999). STEC strains have been observed to endure in fresh ground beef and on fresh leafy green vegetables (Croxen et al. 2013). It has been reported that *stx1* and *stx2*, which are associated with STEC, cause varying degrees and types of tissue damage. Gene *stx2*, in particular, demonstrates greater toxicity to human renal endothelial cells than *stx1* (Louise et al. 1995). On the other hand, ETEC adheres firmly to lettuce and leafy vegetables through flagella (Shaw et al. 2011). The growth of enteric pathogens such as ETEC is notably enhanced in plant tissue with mechanical damage, attributed to the increased availability of nutrients (Kyle et al. 2010). These studies suggest lower levels of detected pathotypes, which may be attributed to the extensive sample size in our study.

In this study, 1206 isolates (726 from summer, 480 from winter) were tested for *E. coli* antibiotic susceptibility. *E. coli* isolates from vegetables, water, and hand swabs collected in the summer displayed high resistance to ampicillin 471/489 (96.32%), 126/127 (99.21%), and 108/110 (98.18%), respectively, and erythromycin 416/489 (85.07%), 115/127 (90.55%), and 85/110 (77.27%), respectively (Table 5). Similarly, winter samples revealed *E. coli* isolates from vegetables, water, and hand swabs to be highly resistant to ampicillin 285/290 (98.28%), 102/103 (99.03%), and 85/87 (97.70%), respectively, and erythromycin 267/290 (92.07%), 98/103 (95.15%), and 68/87 (78.16%), respectively (Table 6). A two‐way ANOVA revealed significant seasonal differences in resistance levels for vegetable samples concerning the tested antibiotics, while water and hand swab samples did not show any significant seasonal variation. Specifically, for the vegetable samples, significant seasonal differences were observed in resistance to amikacin, ampicillin, chloramphenicol, ciprofloxacin, colistin, erythromycin, kanamycin, streptomycin, and tetracycline. Although hand swab samples showed higher mean resistance to some antibiotics, such as amikacin and ciprofloxacin, during summer compared to winter, these differences were not statistically significant.

Amikacin resistance was 67/489 (13.70%) in summer vegetable samples, 19/127 (14.96%) in water, and 11/110 (10.00%) in hand swabs. In winter, resistance increased to 18/290 (20.69%) in vegetables but decreased to 8/103 (7.76%) in water, and 6/87 (6.89%) in hand swabs. In a prior study conducted in Korea, it was observed that the resistance level to amikacin in vegetable samples was low, specifically at 1/28 (3.60%), which is lower than the resistance level observed in our current study (Kim and Woo 2014).

For gentamicin, the resistance levels in vegetable samples were 12/489 (2.45%), 0/127 (0.00%) in water samples, and 5/110 (4.55%) in hand swab samples during the summer collection. Similarly, winter the resistance levels were 12/290 (4.14%) in vegetables, 0/103 (0.00%) in water samples, and 4/87 (4.60%) in hand swabs. Globally varying levels of gentamicin resistance in *E. coli* from vegetables were found. Chittagong reported a significant 4/4 (100.00%) resistance (Nigad Nipa et al. 2011), Korea 12/69 (17.40%) (Kim and Woo 2014), Indonesia 9/36 (25.00%) (Waturangi, Hudiono, and Aliwarga 2019), and the UAE 4/145 (2.75%) (Habib et al. 2023). South Africa noted gentamicin resistance in vegetables below 10.00% (1/67) (Richter et al. 2021). In Portugal, 6/56 (10.70%) resistance in *E. coli* from vegetables to gentamicin was reported (Araújo et al. 2017). In Malaysia, no gentamicin resistance (0/148 (0.00%)) was observed in *E. coli* from food handlers' hand swabs (Tan, Lee, and Mahyudin 2014).

In summer samples, kanamycin resistance levels were 489/288 (16.97%) in vegetables, 22/127 (17.32%) in water, and 6/110 (5.45%) in hand swabs. In winter samples, they were 43/290 (14.83%) in vegetables, 18/103 (17.47%) in water, and 4/87 (4.60%) in hand swabs. A previous study conducted in Dhaka showed thatno kanamycin resistance (0/10 (0.00%)) was found in vegetable *E. coli* (Ahmed et al. 2019). In Indonesia, 14/36 (38.89%) of vegetable and fruit *E. coli* were resistant (Waturangi, Hudiono, and Aliwarga 2019). In Malaysia, 2/7 (28.57%) of *E. coli* from food handlers' hand swabs showed resistance (Tan, Lee, and Mahyudin 2014).

In summer, streptomycin resistance levels were 18/489 (3.68%) for vegetables, 1/127 (0.79%) for washing water, and 0/110 (0.00%) for hand swabs. In winter, levels were 56/290 (19.31%) for vegetables and < 1.00% (1/103) for water and 0.00% (0/87) for hand swabs. In Bangladesh, resistance to streptomycin in vegetable *E. coli* varied from 1/10 (10.00%) to 4/4 (100%) (Ahmed et al. 2019; Nigad Nipa et al. 2011). In Korea, organic farm vegetables had 5/28 (17.90%) resistance, and conventional farm vegetables had 20/69 (29.00%) resistance (Kim and Woo 2014). In Indonesia, fruits and vegetables showed 20/36 (55.56%) resistance (Waturangi, Hudiono, and Aliwarga 2019). Portugal reported a high resistance of 52/56 (93.00%) in vegetable *E. coli* (Araújo et al. 2017), while South Africa found 7/40 (17.50%) resistance (Jongman and Korsten 2016).

In summer, *E. coli* resistance to imipenem was 8/489 (1.64%) in vegetables, 1/127 (0.79%) in vegetable washing water, and 2/110 (1.82%) in hand swabs. In winter, resistance was 7/290 (2.41%) in vegetables, 11/103 (10.68%) in washing water, and 5/87 (5.75%) in hand swabs. The observed resistance levels to imipenem in our study varied between seasons, with higher resistance observed in winter compared to summer across all sample types. In the UAE and Portugal, *E. coli* isolated in vegetables had 0/145 and 0/56 (0.00%) resistance (Araújo et al. 2017; Habib et al. 2023), while in South Africa, it was < 10.00% (1/67) (Richter et al. 2021). In Pakistan, resistance was 4/38 (10.50%) (Shah et al. 2015).

In summer, in our study, *E. coli* isolates showed resistance to meropenem at rates of 4/489 (0.82%) in vegetables, 4/127 (3.15%) in vegetable washing water, and 2/110 (1.82%) in hand swabs. In winter, resistance was observed in 0/290 (0.00%) of vegetables, 4/103 (3.88%) of vegetable washing water, and 1/87 (1.15%) of hand swabs. In Ghana, a study reported high resistance with 55/60 (91.70%) of vegetable *E. coli* being resistant to meropenem (Anokyewaa Appau and Ofori 2024). However, in Nigeria, another study found no resistance (0/64 (0.00%)) in vegetable *E. coli* (Igbinosa et al. 2023).

During summer, *E. coli* isolates exhibited resistance to ampicillin at rates of 471/489 (96.32%) in vegetables, 126/127 (99.21%) in the water used to wash the vegetables, and 108/110 (98.18%) in hand swabs. In winter, ampicillin resistance was observed at 285/290 (98.28%) in vegetable samples, 102/103 (99.03%) in the water used to wash vegetables, and 85/87 (97.70%) in isolates from hand swabs. Previous studies conducted in various locations revealed varying levels of ampicillin resistance in *E. coli* isolates from vegetables. In Chittagong, resistance was found to be 4/4 (100.00%) (Nigad Nipa et al. 2011), while in Dhaka, it was observed at 2/10 (20.00%) (Ahmed et al. 2019). A study in Korea reported 6/69 (8.70%) resistance in conventional farm vegetables and 3/28 (10.70%) in organic farm vegetables (Kim and Woo 2014). In Jakarta, resistance in *E. coli* isolated from fruits and vegetables was 8/36 (22.22%) (Waturangi, Hudiono, and Aliwarga 2019), while in the UAE, it was 30/145 (20.68%) (Habib et al. 2023). South African studies showed resistance rates ranging from 8/40 (20.00%) to 26/67 (38.81%) (Jongman and Korsten 2016; Richter et al. 2021). In Pakistan, *E. coli* isolated from salad vegetables exhibited 33/38 (87.00%) resistance (Shah et al. 2015), while in Ghana and Nigeria, resistance levels were 54/60 (90.00%) (Anokyewaa Appau and Ofori 2024) and 31/64 (48.40%) (Igbinosa et al. 2023), respectively. A study in Malaysia regarding antimicrobial resistance of *E. coli* from food handlers' hands found that 4/7 (57.14%) of isolates were resistant to ampicillin (Tan, Lee, and Mahyudin 2014). From all the previous studies cited, it is evident that the resistance rates of *E. coli* to ampicillin were generally high. However, in our study, the resistance rates appeared to be even higher in both summer and winter seasons.

During both summer and winter collections, resistance levels to ciprofloxacin were low: 36/489 (7.36%) and 23/290 (7.93%) in vegetables, 16/127 (12.6%) and 11/103 (10.68%) in water samples, and 17/110 (15.45%) and 10/87 (11.49%) in hand swabs, respectively. While some studies reported low resistance rates, such as in Dhaka 0/10 (0.00%) (Ahmed et al. 2019) and Chittagong 0/4 (0.00%) (Nigad Nipa et al. 2011), others found slightly higher rates, such as in Jakarta 4/36 (11.11%) (Waturangi, Hudiono, and Aliwarga 2019), the UAE 9/145 (6.20%) (Habib et al. 2023), Portugal 2/56 (3.60%) (Araújo et al. 2017), and South Africa 2/40 (5.00%) (Jongman and Korsten 2016). Notably, studies in Ghana 54/60 (90.00%) (Anokyewaa Appau and Ofori 2024) and Nigeria 31/64 (48.40%) (Igbinosa et al. 2023) reported significantly higher resistance rates. A study conducted in Malaysia on the antimicrobial resistance of *E. coli* from food handlers' hands found that 1/7 (14.29%) of isolates were resistant to ciprofloxacin (Tan, Lee, and Mahyudin 2014). However, the majority of studies, excluding those in Ghana and Nigeria, reported lower resistance levels to ciprofloxacin, consistent with our findings.

In our study, conducted during both summer and winter collections, remarkably high resistance levels of *E. coli* to erythromycin was observed. During the summer, resistance rates in vegetable samples were 416/489 (85.07%), 115/127 (90.55%) in water used to wash vegetables, and 85/110 (77.27%) in hand swab samples. Similarly, in winter samples, resistance levels remained notably elevated, with values of 267/290 (92.07%) in vegetable isolates, 98/103 (95.15%) in water samples, and 68/87 (78.16%) in hand swabs. These findings starkly contrast with previous studies, where resistance percentages were notably lower, such as 5/10 (50.00%) in Dhaka (Ahmed et al. 2019) and 3/4 (75.00%) in Chittagong (Nigad Nipa et al. 2011).

Our study found varying levels of chloramphenicol resistance in *E. coli* isolated from vegetables during summer and winter. In summer, resistance was 50/489 (10.22%) in vegetables, 3/127 (2.36%) in vegetable wash water, and 2/110 (1.82%) in hand swabs. In winter, resistance rose to 47/290 (16.20%) in vegetables, 3/103 (2.91%) in wash water, and 2/87 (2.30%) in hand swabs. Compared to other studies, our findings generally aligned with lower resistance levels reported in Dhaka 0/10 (0.00%) (Ahmed et al. 2019), Korea's organic farms 1/28 (3.60%), conventional farms 1/69 (1.40%) (Kim and Woo 2014), UAE 10/145 (6.89%) (Habib et al. 2023), South Africa 6/70 (8.57%) to 8/67 (11.94%) (Jongman and Korsten 2016; Richter et al. 2021), and Portugal 1/56 (1.80%) (Araújo et al. 2017). However, they contrasted sharply with higher resistance observed in Pakistan 30/38 (79.00%) (Shah et al. 2015), Ghana 52/60 (86.60%) (Anokyewaa Appau and Ofori 2024), and Nigeria 17/64 (26.60%) (Igbinosa et al. 2023). Additionally, a study in Malaysia reported 6/7 (85.71%) resistance among *E. coli* isolates from food handlers' hands, differing significantly from our results (Tan, Lee, and Mahyudin 2014).

Our study observed varying levels of tetracycline resistance in *E. coli* isolated from vegetables, water used for washing the vegetables, and hand swabs during summer and winter. In summer, resistance was 15 /489 (3.06%) in vegetables, 4/127 (3.15%) in vegetable wash water, and 5/110 (4.45%) in hand swabs. In winter, vegetable resistance increased to 18/290 (6.20%), with 3/103 (2.91%) in vegetable wash water and 2/87 (5.75%) in hand swabs. Compared to other studies, our findings generally showed lower resistance levels, which was also reported in a Dhaka study 0/10 (0.00%) (Ahmed et al. 2019). Higher resistance to tetracycline was observed in Korea's organic farms 4/28 (14.30%) and conventional farms 26/69 (37.70%) (Kim and Woo 2014), in Pakistan 35/38 (92.00%) (Shah et al. 2015), Ghana 54/60 (90.00%) (Anokyewaa Appau and Ofori 2024), and Nigeria 33/64 (51.60%) (Igbinosa et al. 2023). Additionally, a study in Malaysia reported 2/7 (28.57%) resistance among *E. coli* isolates from food handlers' hands, differing significantly from our results (Tan, Lee, and Mahyudin 2014).

Our study found varying levels of colistin resistance in *E. coli* isolated from vegetables, water used to wash vegetables, and hand swabs during both summer and winter seasons. In summer, resistance rates were 113/489 (23.11%) in vegetables, 22/127 (17.32%) in wash water, and 17/110 (15.45%) in hand swabs. In winter, resistance rates were 68/290 (23.45%) in vegetables, 14/103 (13.59%) in wash water, and 8/87 (9.20%) in hand swabs. This contrasts with previous studies; one in Malaysia (Aklilu and Raman 2020) reported 1/6 (1.67%) resistance in vegetables, while another in India found only 2/73 (2.70%) resistance in fruits and vegetables (Verma et al. 2018). Our study indicates notably higher resistance levels. A detailed overall resistance of *E. coli* to various antibiotics is detailed in Figure 2.

Our findings contrast with several studies conducted in different regions. These discrepancies may stem from various factors such as differences in geographical locations, sample collection methods, bacterial strains, or antibiotic usage patterns.

In our study, we observed that during the summer, *E. coli* isolated from spring onion samples exhibited the highest percentage of MDR at 30/53 (56.60%) (Table 7), while during winter, *E. coli* isolated from carrot samples showed the highest percentage at 23/32 (71.87%) (Table 7). These findings align with similar studies conducted globally. In Dhaka, Bangladesh, 9/10 (90.00%) of *E. coli* isolated from vegetables were multidrug resistant (Ahmed et al. 2019), while in Chittagong, this figure was 3/4 (75.00%) (Nigad Nipa et al. 2011). In Jakarta, Indonesia, a study reported 30/36 (83.33%) MDR among *E. coli* isolates from vegetables and fruits (Waturangi, Hudiono, and Aliwarga 2019), while a study in the UAE found 20/145 (13.79%) of *E. coli* isolates from locally sourced leafy salad vegetables to be multidrug resistant (Habib et al. 2023). A South African study reported that 27/67 (40.30%) of *E. coli* isolates exhibited MDR (Richter et al. 2021), and a study in Portugal observed an 8/38 (21.00%) MDR level among *E. coli* isolates from vegetables (Araújo et al. 2017). Studies in Ghana and Nigeria reported high levels of MDR, with 58/60 (96.00%) (Anokyewaa Appau and Ofori 2024) and 55/64 (85.90%) (Igbinosa et al. 2023) of *E. coli* isolates from vegetables exhibiting resistance, respectively. In an Indian study, 6/73 (10.70%) of *E. coli* isolates from fruits and vegetables were found to be multidrug resistant (Verma et al. 2018), while a Malaysian study observed 6/7 (85.71%) MDR among *E. coli* isolates from hand swabs (Tan, Lee, and Mahyudin 2014). These findings collectively highlight a global concern regarding the prevalence of multidrug‐resistant *E. coli* in vegetables, emphasizing the need for comprehensive strategies to address this issue.

XDR isolates were found in mint 1/46 (2.17%) and cabbage 1/26 (1.96%) samples that were collected in summer (Table 7). Additionally, XDR isolates (2/32 (6.25%)) were observed in carrot samples, which were collected in winter (Table 7). In a previous study conducted in Thailand, it was observed that 11/96 (11.50%) of *E. coli* isolates from vegetables were XDR (Datta et al. 2024), which did not agree with our findings.

## 5 Conclusions

This extensive study conducted in Bangladesh highlighted concerning levels of *E. coli* contamination in vegetables, water used for washing produce, and hand swab samples. A two‐way ANOVA found no significant difference in *E. coli* prevalence between the two seasons. However, seasonal effects were noted, with significant differences in *E. coli* pathotype prevalence across all sample types except for *CVD432*, consistently showing higher levels in summer. While no significant seasonal differences were found for the *eaeA*_*O157*_, *stx2, eaeA, ipaH*, and *eltB* genes among vegetable samples, the *stx1* gene did exhibit higher prevalence in summer for all the vegetable samples. Additionally, the analysis indicated significant seasonal differences in resistance levels for vegetable and water samples against the tested antibiotics, with significant variations for amikacin, ampicillin, chloramphenicol, ciprofloxacin, colistin, erythromycin, kanamycin, streptomycin, and tetracycline in vegetables and for imipenem and kanamycin in water samples. In contrast, no significant differences were observed in hand swab samples for any antibiotics between summer and winter, although amikacin and ciprofloxacin showed higher mean resistance in summer, which was not statistically significant. Elevated resistance to antibiotics such as ampicillin and erythromycin was observed across seasons. Improvements in food safety practices are necessary, emphasizing proper handling from cultivation to post‐harvest stages. Additionally, increased awareness among consumers and regulatory authorities regarding the risks associated with contaminated vegetables and the emergence of antibiotic‐resistant strains is imperative to safeguard public health and mitigate the spread of foodborne illnesses in Bangladesh.

## Author Contributions

**Maftuha Ahmad Zahra:** data curation (equal), formal analysis (equal), investigation (equal), methodology (equal), validation (equal), writing – original draft (lead), writing – review and editing (lead). **Golam Niaj Murshidi:** data curation (equal), formal analysis (equal), investigation (equal), methodology (equal), validation (equal), visualization (equal), writing – original draft (supporting). **Unmilita Das Moon:** data curation (equal), formal analysis (equal), investigation (equal), methodology (equal), validation (equal), writing – original draft (supporting). **Sumaiya Sultana:** methodology (equal), validation (equal). **Fahim Kabir Monjurul Haque:** conceptualization (lead), funding acquisition (lead), methodology (equal), project administration (lead), resources (lead), supervision (lead), writing – original draft (supporting), writing – review and editing (supporting).

## Ethics Statement

The study was conducted in accordance with the Declaration of Helsinki, and ethical permission was obtained from the Institutional Review Board of BRAC University. After the study was explained to the vendors, their written consent was collected prior to sample collection. For the convenience of the participants, the consent form was prepared in Bengali.

## Consent

Written informed consent was obtained from all study participants.

## Conflicts of Interest

The authors declare no conflicts of interest.

## Supporting information


Data S1.

## Data Availability

All relevant data are within the manuscript and its supporting information.
