# Supplementary material for: Seasonal Analysis of Pathogenic Escherichia coli Contamination in Vegetables, Washing Water, and Vendor Hygiene: Virulence Group Classification and Antibiotic Resistance
Source: Food Sci Nutr. 2025 Jan 9;13(1):e4723. doi: 10.1002/fsn3.4723 (PMC11758462; doi:10.1002/fsn3.4723)
Supplement: Supplementary file 1 — Data S1. [file FSN3-13-e4723-s001.docx]

**Supplementary Materials**

**Supplementary Table 1: Areas of Sample Collection**

| 1. Mohakhali | 8. Dhanmondi |
| --- | --- |
| 1. Rampura | 9. Bashundhara |
| 1. Gulshan-1 | 10. Rajarbag |
| 1. Gulshan-2 | 11. Uttara |
| 1. Mirpur | 12. Farmgate |
| 1. Banani | 13. Motijheel |
| 1. Khilgaon | 14. Mohammadpur |

**Supplementary Table 2:** **Number of** **Vegetable Samples Collected**

| **Name** | **Scientific Name** | **Samples Collected in Winter** | **Samples Collected in Summer** | **Total samples** |
| --- | --- | --- | --- | --- |
| Tomato | *Lycopersican esculentum* | 25 | 37 | 62 |
| Carrot | *Daucas carota* | 37 | 40 | 77 |
| Capsicum | *Capsicum Fruits scence* | 27 | 22 | 49 |
| Cucumber | *Cucumis sativas* | 22 | 34 | 56 |
| Coriander | *Coriandrum sativum* | 21 | 56 | 77 |
| Lettuce | *Lactuca sativa* | 24 | 41 | 65 |
| Mint (Pudina) | *Mentha arvensis* | 22 | 47 | 69 |
| Green chilli | *Capsicum annuum* | 19 | 58 | 77 |
| Cabbage | *Brassica oleracea var. capitata* | 25 | 29 | 54 |
| Spring onion | *Allium fistulosum* | 28 | 26 | 54 |
| Total |  | 250 | 390 | 640 |

**Supplementary Table 3: Colony Morphology of *Escherichia coli* on Selective Media**

| **Organism** | **Media** | **Expected Colony Morphology** |
| --- | --- | --- |
| *Escherichia coli* | MacConkey Agar | Non-mucoid red/pink colonies |
| *Escherichia coli* | EMB Agar | Purple with a black center and green metallic sheen |

**Supplementary Table 4: Biochemical Test Interpretation for *Escherichia coli* isolates**

| **Organism** | **Catalase** | **Oxidase** | **Glucose**  **Ferm.** | **Sucrose**  **Ferm.** | **Lactose**  **Ferm.** | **Gas Prod.** | **H_2_S**  **Prod.** | **Motility** | **Indole** | **Urease** | **MR** | **VP** | **Citrate utilization** |
| --- | --- | --- | --- | --- | --- | --- | --- | --- | --- | --- | --- | --- | --- |
| *Escherichia coli* | +ve | -ve | +ve | variable | +ve | +ve | -ve | +ve | +ve | -ve | +ve | -ve | -ve |

**Supplementary Table 5: Primers and PCR Conditions used in this Study**

| **Target Gene** | **Primers** | **Sequence** | **Amplicon Size** | **Conditions** | **Reference** |
| --- | --- | --- | --- | --- | --- |
| 16S rRNA gene | ECO-1 | 5'- GACCTCGGTTTAGTTCACAGA-3' | 585 bp | The PCR conditions were: 1 cycle of 95°C for 7 min, 35 cycles of 94°C for 1 min, 55°C for 1 min and 72°C for 1 min and 1 cycle of 72°C for 7 min. | (Conte et al., 2006) |
|  | ECO-2 | 5'-CACACGCTGACGCTGACCA-3' |  |  |  |
| *eaeA_O15_*_7_ | EAE157-F | 5'- CAGGTCGTCGTGTCTGCTAAA-3' | 1087 bp | The PCR conditions were: 15 s at 94°C, followed by 15 s at 65°C and 75 s at 72°C for 35 cycles and a final extension for 5 min at 72°C. | (Gannon et al., 1997) |
|  | EAE157-R | 5'-TCAGCGTGGTTGGATCAACCT-3' |  |  |  |
| *Stx* (STEC /Shiga Toxin) | *stx1* | F- 5'- ATAAATCGCCATTCGTTGACTAC -3' | 180 bp | The conditions involved 35 cycles with denaturation at 95°C for 1 min, annealing at 65°C for 2 min for the initial 10 cycles, decreasing to 60°C by cycle 15, and elongation at 72°C for 1.5 min, increasing to 2.5 min from cycles 25 to 35. | (Paton & Paton, 1998) |
|  |  | R -5'- AGAACGCCCACTGAGATCATC -3' |  |  |  |
|  | *stx2* | F-5'- GGCACTGTCTGAAACTGCTCC -3' | 255 bp |  |  |
|  |  | R-5'-TCGCCAGTTATCTGACATTCTG -3' |  |  |  |
| *EaeA*  (EPEC) | *eaeA* | F-5'- TGCGGCACAACAGGCGGCGA-3' | 629 bp | Initial denaturation at 95°C for 5 min followed by 30 cycles at 95°C for 60s 67°C for 120s and 72°C for 60s and final extension at 72°C for 5 min. | (Surendraraj, Thampuran, & Joseph, 2010) |
|  |  | R-5'-CGGTCGCCGCACCAGGATTC-3' |  |  |  |
| *IpaH*  (EIEC) | *ipaH* | F- 5’-AGGTTAATCT-TTGCAGGGCT-3’ | 423 bp | The PCR protocol included an initial denaturation at 95°C for 3 min, followed by 35 cycles of denaturation at 94°C for 1 min, annealing at 55°C for 1 min, and extension at 72°C for 1 min with a final extension at 72°C for 10 min. | (Akhter et al., 2012) |
|  |  | R-5’-CAACAACCAGCTTACTGCCT -3’ |  |  |  |
| *EltB*  (ETEC) | *eltB* | F-5'-TCTCTATGTGCATACGGAGC-3' | 322 bp | The PCR conditions were 96°C for 4 min, 94°C for 20 s, 55°C for 20 s, and 72°C for 10 s for 30 cycles, with a final 7-min extension at 72°C. | (Nguye, Le Van, Le Huy, Gia, & Weintrub, 2005) |
|  |  | R-5'-CCATACTGATTGCCGCAAT-3' |  |  |  |
| *CVD 432*  (EAEC) | *CVD 432* | F-5'-CTGGCGAAAGACTGTATCAT-3' | 630 bp | The PCR conditions were 5 min at 94ºC; 45 sec at 94°C; 30 sec at 54°C; 45 sec at 72ºC; 10 min at 72°C; 40 cycles | (Aranda, Fagundes-Neto, & Scaletsky, 2004) |
|  |  | R-5'-CAATGTATAGAAATCCGCTGTT-3**'** |  |  |  |


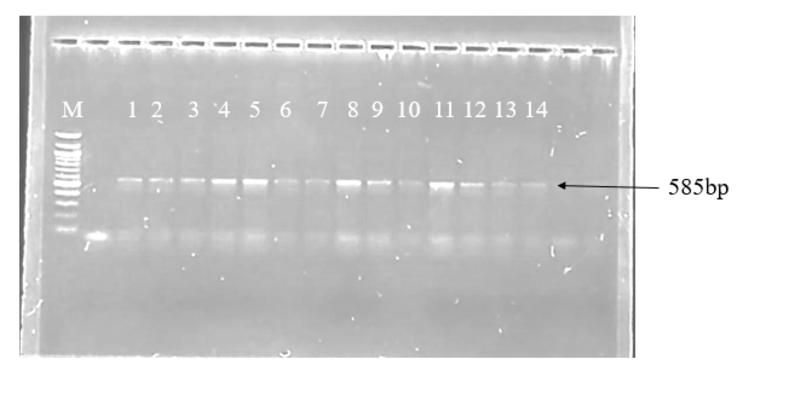


**Supplementary Figure 1: Agarose gel electrophoresis of PCR assay of *Escherichia coli* isolates**. Here, Lane M is 100 bp DNA marker, and Lane (1-14) are some positive samples at 585 bp. Lane 8 served as the positive control.


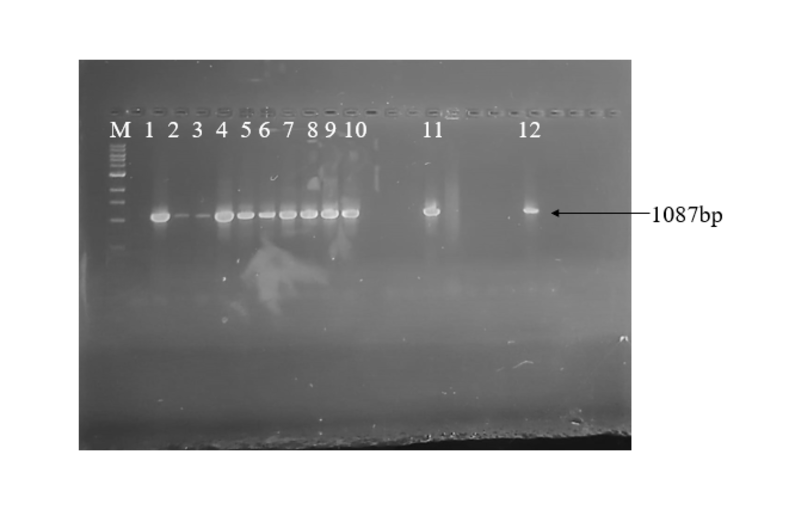


**Supplementary Figure 2:**  **Agarose gel electrophoresis of PCR assay of *eaeA_O157_* gene.** Here, Lane M is 1kb DNA marker, and Lane (1-12) are some positive samples at 1087bp.


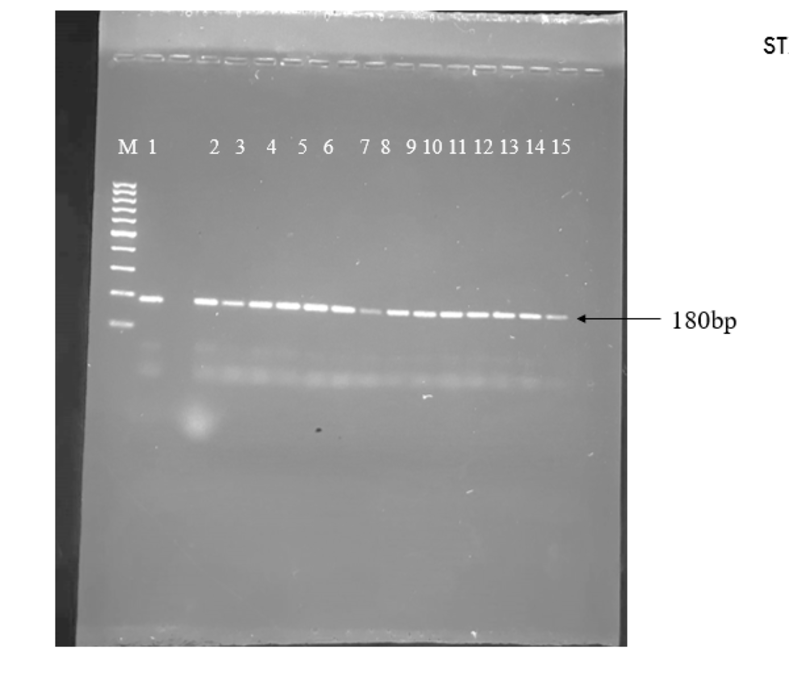


**Supplementary Figure 3:** **Agarose gel electrophoresis of PCR assay of *stx1* gene.** Here, Lane M is 100 bp DNA marker, and Lane (1-15) are some positive samples at 180bp.


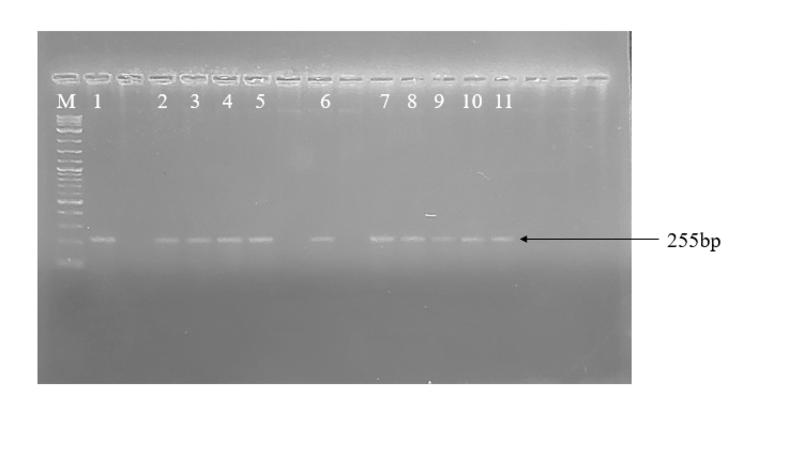


**Supplementary Figure 4: Agarose gel electrophoresis of PCR assay of *stx2* gene.** Here Lane M is 100bp DNA marker, and Lane (1-11) are some positive samples at 255bp.


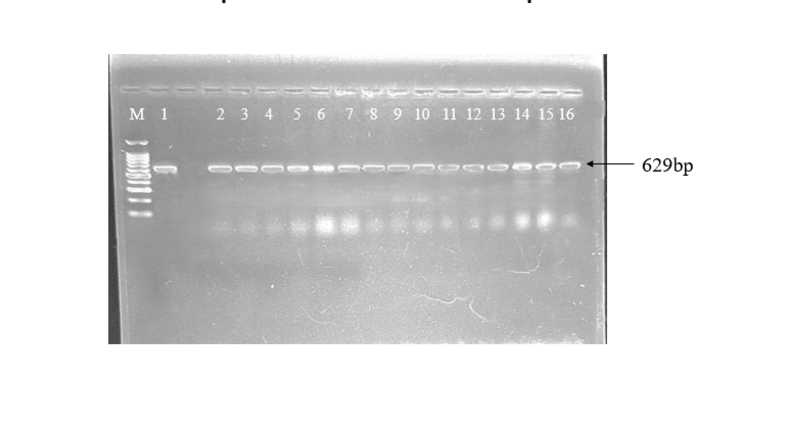


**Supplementary Figure 5:**  **Agarose gel electrophoresis of PCR assay of *eaeA* gene.** Here, Lane M is 100bp DNA marker, and Lane (1-16) are some positive samples at 629bp.


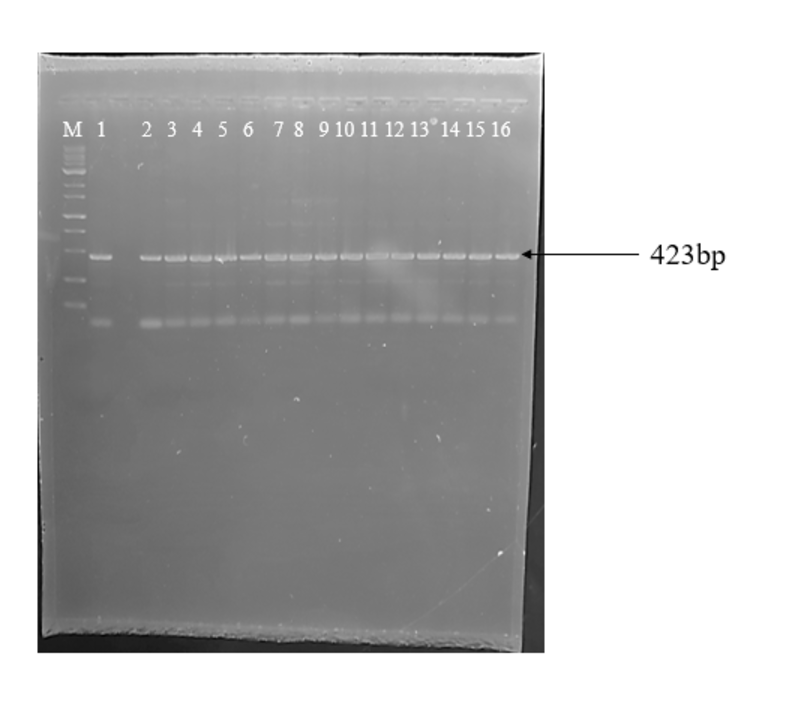


**Supplementary Figure 6:** **Agarose gel electrophoresis of PCR assay of *ipaH* gene.** Here, Lane M is 1kb DNA marker, and Lane (1-16) are some positive samples at 423bp.


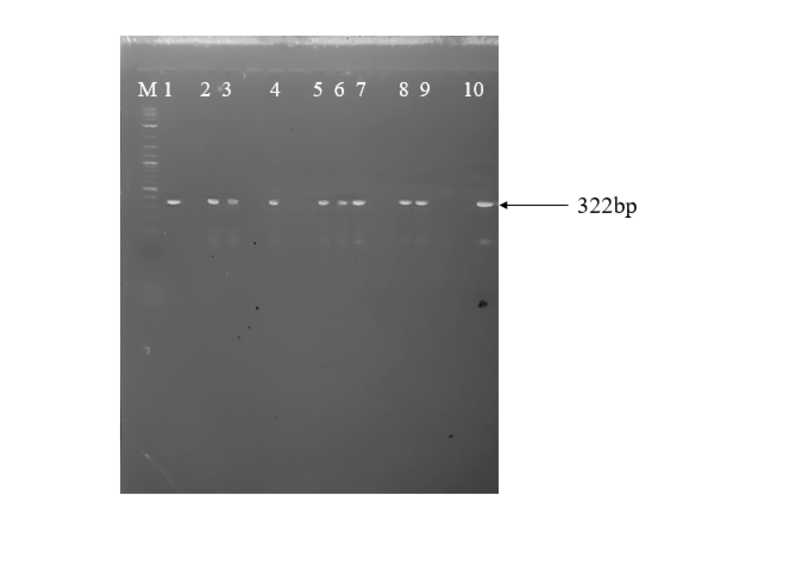


**Supplementary Figure 7:**  **Agarose gel electrophoresis of PCR assay of *eltB* gene.** Here, Lane M is 100bp DNA marker, and Lane (1-10) are some positive samples at 322bp.


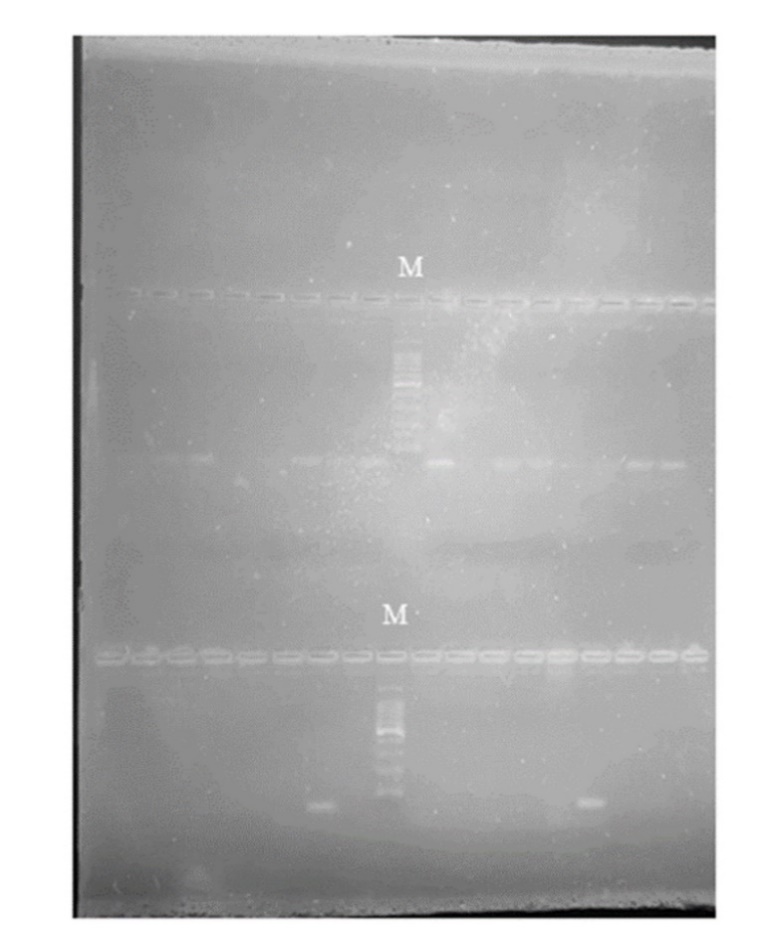


**Supplementary Figure 8:**  **Agarose gel electrophoresis of PCR assay of *CVD432* gene.** Here, Lane M is 100bp DNA marker, and no bands showed the correct band size of 630bp and therefore not included in our positive result.

**Supplementary Table 6: List of Antibiotics Used in This Study**

| **Antibiotic Group** | **Antibiotic Name** | **Disc code** | **Disc potency (µg)** | **Sensitive (mm)** | **Intermediate (mm)** | **Resistant (mm)** |
| --- | --- | --- | --- | --- | --- | --- |
| Aminoglycosides | Amikacin | AK | 30 | ≥ 17 | 15-16 | ≤ 14 |
|  | Gentamicin | GEN | 10 | ≥ 15 | 13-14 | ≤ 12 |
|  | Kanamycin | KAN | 30 | ≥ 18 | 14-17 | ≤ 13 |
|  | Streptomycin | S | 10 | ≥ 15 | 12-14 | ≤ 11 |
| Carbapenem | Imipenem | IMI | 10 | ≥ 23 | 18-22 | ≤ 19 |
|  | Meropenem | MEM | 10 | ≥ 23 | 18-22 | ≤ 19 |
| β-lactam | Ampicillin | AMP | 10 | ≥ 17 | 12-16 | ≤ 13 |
| Fluoroquinolone | Ciprofloxacin | CIP | 5 | ≥ 21 | 16-20 | ≤ 15 |
| Macrolides | Erythromycin | E | 15 | ≥ 23 | 14-22 | ≤ 13 |
| Phenicol | Chloramphenicol | C | 30 | ≥ 18 | 13-17 | ≤ 12 |
| Polymyxin | Colistin | CT | 10 | ≥ 17 | 12-16 | ≤ 11 |
| Tetracycline | Tetracycline | TE | 30 | ≥ 15 | 12-14 | ≤ 11 |

**Supplementary Table 7:** ***Escherichia coli* Isolates Categorized Based on Virulence Factors Identified in Vegetable Samples Collected in Summer.** Here n indicates the number of positive isolates listed outside the parentheses, with the corresponding percentage given inside.

| **Group** | **Targeted Genes** | **Summer Samples of Vegetables Containing *E. coli***  **Number of isolates (%)** | | | | | | | | | |
| --- | --- | --- | --- | --- | --- | --- | --- | --- | --- | --- | --- |
|  |  | **Lettuce**  **(n=41)** | **Tomato (n=36)** | **Capsicum (n=20)** | **Cucumber**  **(n=31)** | **Cabbage (n=27)** | **Spring Onion**  **(n=26)** | **Carrot (n=39)** | **Coriander**  **(n=56)** | **Mint (n=46)** | **Green**  **Chili (n=57)** |
| *E. coli* O157:H7 strain | *eaeA_O157_* | 3  (7.31%) | 2  (5.55%) | 3  (15.00%) | 4  (12.90%) | 4  (14.81%) | 5  (19.23%) | 3  (7.69%) | 2  (3.57%) | 5  (10.86%) | 4  (7.01%) |
| STEC | *stx1* | 8  (19.51%) | 9 (25.00%) | 5  (25.00%) | 7  (22.58%) | 6  (22.22%) | 8  (30.76%) | 9  (23.07) | 5  (8.92%) | 8  (17.39%) | 8  (14.03%) |
|  | *stx2* | 9  (21.95%) | 5  (13.88%) | 8  (40.00%) | 6  (19.35%) | 3  (1.11%) | 8  (30.76%) | 5  (12.82%) | 9  (16.07%) | 9  (19.56%) | 7  (12.28%) |
| EPEC | *eaeA* | 3  (7.31%) | 3  (8.33%) | 4  (20.00%) | 5  (16.12%) | 5  (18.51%) | 3  (11.53%) | 5  (12.82%) | 3  (5.35%) | 5  (10.86%) | 4  (7.01%) |
| EIEC | *ipaH* | 7  (17.07%) | 8  (22.22%) | 7  (35.00%) | 7  (22.58%) | 5  (18.51%) | 6  (23.07%) | 7  (17.94%) | 4  (7.14%) | 3  (6.52%) | 5  (8.77%) |
| ETEC | *eltB* | 5  (12.19%) | 3  (8.33%) | 4  (20.00%) | 4  (12.90%) | 5  (18.51%) | 6  (23.07%) | 6  (15.38%) | 4  (7.14%) | 4  (8.69%) | 3  (5.26%) |
| EAEC | *CVD432* | 0(0.00%) | 0  (0.00%) | 0  (0.00%) | 0  (0.00%) | 0  (0.00%) | 0  (0.00%) | 0  (0.00%) | 0  (0.00%) | 0  (0.00%) | 0 (0.00%) |

**Supplementary Table 8: *Escherichia coli* Isolates Categorized Based on Virulence Factors Identified in Vegetable Samples Collected in Winter.** Here n indicates the number of positive isolates listed outside the parentheses, with the corresponding percentage given inside.

| **Group** | **Targeted Genes** | **Vegetable Samples collected in Winter**  **Number of isolates (%)** | | | | | | | | | |
| --- | --- | --- | --- | --- | --- | --- | --- | --- | --- | --- | --- |
|  |  | **Lettuce**  **(n=24)** | **Tomato (n=25)** | **Capsicum (n=25)** | **Cucumber**  **(n=21)** | **Cabbage (n=25)** | **Spring**  **Onion**  **(n=26)** | **Carrot (n=35)** | **Coriander**  **(n=21)** | **Mint (n=22)** | **Green chili (n=19)** |
| *E. coli* O157:H7 strain | *eaeA_O157_* | 2  (8.33%) | 2  (8.00%) | 2  (8.00%) | 1  (4.76%) | 1  (4.00%) | 2  (7.69%) | 2  (5.71%) | 3  (14.28%) | 2  (9.09%) | 1  (5.26%) |
| STEC | *stx1* | 2  (8.33%) | 4  (16.00%) | 3  (12.00%) | 4  (19.04%) | 2  (8.00%) | 1  (3.84%) | 3  (8.57%) | 3  (14.28%) | 3  (13.63%) | 2  (10.52%) |
|  | *stx2* | 3  (12.50%) | 4  (16.00%) | 4  (16.00%) | 3  (14.28%) | 3  (12.00%) | 2  (7.69%) | 3  (8.57%) | 3  (14.28%) | 2  (9.09%) | 3  (15.78%) |
| EPEC | *eaeA* | 2  (8.33%) | 3  (12.00%) | 2  (8.00%) | 1  (4.76%) | 2  (8.00%) | 1  (3.84%) | 4  (11.42%) | 2  (9.52%) | 2  (9.09%) | 2  (10.52%) |
| EIEC | *ipaH* | 3  (12.50%) | 3  (12.00%) | 3  (12.00%) | 2  (9.52%) | 2  (8.00%) | 2  (7.69%) | 3  (8.57%) | 3  (14.28%) | 3  (13.63%) | 2  (10.52%) |
| ETEC | *eltB* | 2  (8.33%) | 4  (16.00%) | 2  (8.00%) | 1  (4.76%) | 1  (4.00%) | 2  (7.69%) | 2  (5.71%) | 2  (9.52%) | 2  (9.09%) | 2  (10.52%) |
| EAEC | *CVD432* | 0  (0.00%) | 0  (0.00%) | 0  (0.00%) | 0  (0.00%) | 0  (0.00%) | 0  (0.00%) | 0  (0.00%) | 0  (0.00%) | 0 (0.00%) | 0  (0.00%) |

**Supplementary Table 9: Area-Wise Distribution of Vegetable Samples Collected in Summer and Counts of MDR and XDR Isolates in Dhaka City.** Values outside parentheses represent the total MDR and XDR isolates for each area, while values in parentheses specify the separate counts of MDR and XDR isolates, respectively.

| **Area Code** | **Area Name** | **Vegetable Samples** | | | **Water Samples** | | | **Hand Swabs** | | | **Area-wise Total Isolates No. (MDR +XDR Isolates No.)** |
| --- | --- | --- | --- | --- | --- | --- | --- | --- | --- | --- | --- |
|  |  | Total Isolates | MDR No. | XDR No. | Total Isolates | MDR No. | XDR No. | Total Isolates | MDR No. | XDR No. |  |
| 123 | Mohakhali | 35 | 22 | 0 | 12 | 5 | 0 | 6 | 0 | 0 | 53 (27+0) |
| 124 | Rampura | 31 | 14 | 0 | 9 | 5 | 0 | 8 | 2 | 0 | 48 (21+0) |
| 125 | Gulshan-1 | 32 | 22 | 0 | 8 | 7 | 0 | 8 | 8 | 0 | 48 (37+0) |
| 126 | Mirpur | 40 | 27 | 0 | 12 | 5 | 0 | 12 | 11 | 0 | 64 (43+0) |
| 127 | Gulshan-2 | 37 | 30 | 1 | 12 | 11 | 0 | 12 | 11 | 0 | 61 (52+1) |
| 128 | Banani | 33 | 9 | 0 | 12 | 1 | 0 | 9 | 1 | 0 | 54 (11+0) |
| 129 | Khilgaon | 31 | 4 | 0 | 12 | 5 | 0 | 12 | 3 | 0 | 55 (12+0) |
| 130 | Dhanmondi | 35 | 1 | 0 | 7 | 0 | 0 | 10 | 0 | 0 | 52 (1+0) |
| 131 | Bashundhara | 29 | 4 | 0 | 8 | 2 | 0 | 4 | 0 | 0 | 41 (6+0) |
| 132 | Rajarbag | 40 | 5 | 0 | 3 | 0 | 0 | 12 | 1 | 0 | 55 (6+0) |
| 133 | Uttara | 40 | 6 | 0 | 11 | 0 | 0 | 8 | 0 | 0 | 59 (6+0) |
| 134 | Farmgate | 37 | 24 | 0 | 3 | 2 | 0 | 5 | 4 | 0 | 45 (30+0) |
| 135 | Motijheel | 38 | 33 | 1 | 8 | 7 | 0 | 0 | 0 | 0 | 46 (40+1) |
| 136 | Mohammadpur | 31 | 17 | 0 | 10 | 10 | 0 | 4 | 0 | 0 | 45 (27+0) |
|  | Total | 489 | 218 | 2 | 127 | 60 | 0 | 110 | 41 | 0 | 726 (319+2) |

**Supplementary Table 10: Area-Wise Distribution of Vegetable Samples Collected in Winter and Counts of MDR and XDR Isolates in Dhaka City.** Values outside parentheses represent the total MDR and XDR isolates for each area, while values in parentheses specify the separate counts of MDR and XDR isolates, respectively.

| **Area Code** | **Area Name** | **Vegetable Samples** | | | **Water Samples** | | | **Hand Swabs** | | | **Area-wise Total Isolates No. (MDR +XDR Isolates No.)** |
| --- | --- | --- | --- | --- | --- | --- | --- | --- | --- | --- | --- |
|  |  | Total Isolates | MDR No. | XDR No. | Total Isolates | MDR No. | XDR No. | Total Isolates | MDR No. | XDR No. |  |
| 11 | Gulshan-1 | 20 | 11 | 0 | 7 | 3 | 0 | 4 | 0 | 0 | 31 (14+0) |
| 12 | Banani | 20 | 12 | 0 | 5 | 3 | 0 | 3 | 0 | 0 | 28 (15+0) |
| 13 | Mohakhali | 20 | 11 | 0 | 5 | 4 | 0 | 4 | 2 | 0 | 29 (17+0) |
| 14 | Farmgate | 20 | 12 | 0 | 8 | 5 | 0 | 5 | 3 | 0 | 33 (20+0) |
| 15 | Rampura | 16 | 12 | 2 | 8 | 8 | 0 | 4 | 4 | 0 | 28 (24+2) |
| 16 | Khilgaon | 24 | 13 | 0 | 12 | 1 | 0 | 6 | 6 | 0 | 42 (20+0) |
| 17 | Dhanmondi | 20 | 9 | 0 | 12 | 5 | 0 | 5 | 4 | 0 | 37 (18+0) |
| 18 | Gulshan-2 | 19 | 9 | 0 | 7 | 0 | 0 | 7 | 1 | 0 | 33 (10+0) |
| 19 | Mohammadpur | 25 | 11 | 0 | 8 | 2 | 0 | 10 | 3 | 0 | 43 (16+0) |
| 20 | Uttara | 16 | 10 | 0 | 3 | 0 | 0 | 10 | 0 | 0 | 29 (10+0) |
| 21 | Mirpur | 25 | 11 | 0 | 11 | 0 | 0 | 4 | 0 | 0 | 40 (11+0) |
| 22 | Bashundhara | 20 | 14 | 0 | 3 | 3 | 0 | 12 | 1 | 0 | 35 (18+0) |
| 23 | Motijheel | 23 | 13 | 0 | 6 | 6 | 0 | 8 | 0 | 0 | 37 (19 +0) |
| 24 | Rajarbag | 22 | 9 | 0 | 8 | 6 | 0 | 5 | 4 | 0 | 35 (19+0) |
|  | Total | 290 | 157 | 2 | 103 | 46 | 0 | 87 | 28 | 0 | 480 (231+2) |

**References:**

Akhter, F., Fakruddin, M., Azmuda, N., Begum, A., Birkeland, N. K., SKhan, S. I., & Akhter, H. (2012). Detection of ipaH gene in Large Plasmid of Escherichia coli Isolated from Different Sources of Bangladesh. *Bangladesh Journal of Microbiology*, *28*(2), 84–87. https://doi.org/10.3329/bjm.v28i2.11822

Aranda, K. R. S., Fagundes-Neto, U., & Scaletsky, I. C. A. (2004). Evaluation of Multiplex PCRs for Diagnosis of Infection with Diarrheagenic Escherichia coli and Shigella spp. *Journal of Clinical Microbiology*, *42*(12), 5849–5853. https://doi.org/10.1128/JCM.42.12.5849-5853.2004

Conte, M. P., Schippa, S., Zamboni, I., Penta, M., Chiarini, F., Seganti, L., … Cucchiara, S. (2006). Gut-associated bacterial microbiota in paediatric patients with inflammatory bowel disease. *Gut*, *55*(12), 1760–1767. https://doi.org/10.1136/gut.2005.078824

Gannon, V. P. J., D’Souza, S., Graham, T., King, R. K., Rahn, K., & Read, S. (1997). Use of the flagellar H7 gene as a target in multiplex PCR assays and improved specificity in identification of enterohemorrhagic Escherichia coli strains. *Journal of Clinical Microbiology*, *35*(3), 656–662. https://doi.org/10.1128/jcm.35.3.656-662.1997

Nguyen, T. V., Le Van, P., Le Huy, C., Gia, K. N., & Weintraub, A. (2005). Detection and Characterization of Diarrheagenic Escherichia coli from Young Children in Hanoi, Vietnam. *Journal of Clinical Microbiology*, *43*(2), 755–760. https://doi.org/10.1128/JCM.43.2.755-760.2005

Paton, A. W., & Paton, J. C. (1998). Detection and Characterization of Shiga Toxigenic Escherichia coli by Using Multiplex PCR Assays for stx 1 , stx 2 , eaeA , Enterohemorrhagic E. coli hlyA , rfb O111 , and rfb O157. *Journal of Clinical Microbiology*, *36*(2), 598–602. https://doi.org/10.1128/JCM.36.2.598-602.1998

Surendraraj, A., Thampuran, N., & Joseph, T. C. (2010). Molecular Screening, Isolation, and Characterization of Enterohemorrhagic Escherichia coli O157:H7 from Retail Shrimp. *Journal of Food Protection*, *73*(1), 97–103. https://doi.org/10.4315/0362-028x-73_1_97
